# Structure and Reactivity of Oxygen-Bridged Diamino Dicopper(II) Complexes in Cu-Ion-Exchanged Chabazite Catalyst for NH_3_-Mediated Selective Catalytic Reduction

**DOI:** 10.1021/jacs.0c06270

**Published:** 2020-08-24

**Authors:** Chiara Negri, Tommaso Selleri, Elisa Borfecchia, Andrea Martini, Kirill A. Lomachenko, Ton V. W. Janssens, Michele Cutini, Silvia Bordiga, Gloria Berlier

**Affiliations:** † Department of Chemistry and NIS Centre, University of Turin, Via Giuria 7, I-10125 Turin, Italy; ‡ Dipartimento di Energia, Laboratorio di Catalisi e Processi Catalitici, Politecnico di Milano, Via La Masa 34, I-20156 Milano, Italy; § Smart Materials Research Institute, Southern Federal University, Sladkova Street 174/28, 344090 Rostov-on-Don, Russia; ∥ European Synchrotron Radiation Facility, 71 Avenue des Martyrs, CS 40220, 38043 Grenoble Cedex 9, France; ⊥ Umicore Denmark ApS, Kogle Allé 1, 2970 Hørsholm, Denmark

## Abstract

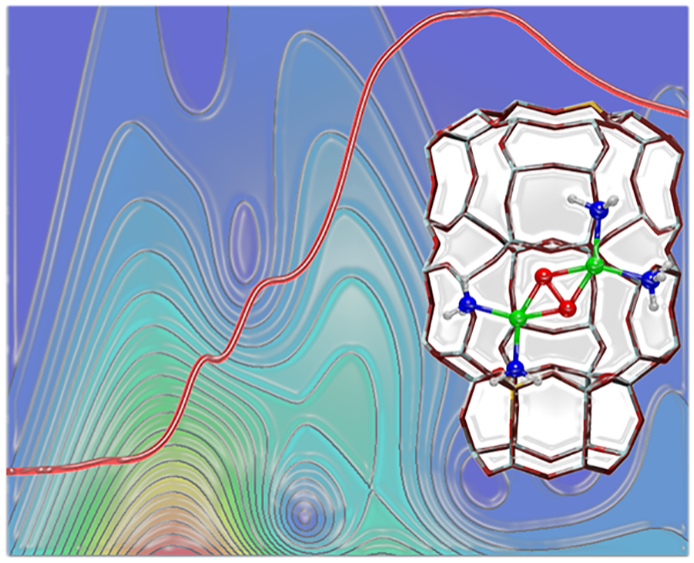

The NH_3_-mediated selective catalytic reduction (NH_3_-SCR) of NOx over Cu-ion-exchanged chabazite (Cu-CHA) catalysts is the basis of the technology for abatement of NOx from diesel vehicles. A crucial step in this reaction is the activation of oxygen. Under conditions for low-temperature NH_3_-SCR, oxygen only reacts with Cu^I^ ions, which are present as mobile Cu^I^ diamine complexes [Cu^I^(NH_3_)_2_]^+^. To determine the structure and reactivity of the species formed by oxidation of these Cu^I^ diamine complexes with oxygen at 200 °C, we have followed this reaction, using a Cu-CHA catalyst with a Si/Al ratio of 15 and 2.6 wt% Cu, by X-ray absorption spectroscopies (XANES and EXAFS) and diffuse reflectance UV-Vis spectroscopy, with the support of DFT calculations and advanced EXAFS wavelet transform analysis. The results provide unprecedented direct evidence for the formation of a [Cu_2_(NH_3_)_4_O_2_]^2+^ mobile complex with a side-on μ-η^2^,η^2^-peroxo diamino dicopper(II) structure, accounting for 80–90% of the total Cu content. These [Cu_2_(NH_3_)_4_O_2_]^2+^ are completely reduced to [Cu^I^(NH_3_)_2_]^+^ at 200 °C in a mixture of NO and NH_3_. Some N_2_ is formed as well, which suggests the role of the dimeric complexes in the low-temperature NH_3_-SCR reaction. The reaction of [Cu_2_(NH_3_)_4_O_2_]^2+^ complexes with NH_3_ leads to a partial reduction of the Cu without any formation of N_2_. The reaction with NO results in an almost complete reduction to Cu^I^, under the formation of N_2_. This indicates that the low-temperature NH_3_-SCR reaction proceeds via a reaction of these complexes with NO.

## 1 Introduction

The selective catalytic reduction of NO_*x*_ by ammonia (NH_3_-SCR) to nitrogen and water is the basis for the current technology for NO_*x*_ abatement in the exhaust of lean-burn heavy-duty and passenger vehicles. This technology has already resulted in significant improvements of exhaust gas emissions from diesel vehicles. Catalysts based on Cu-ion exchanged chabazite (Cu-CHA) are very effective for this reaction and are commonly applied today. These catalysts feature a high activity around 200 °C, a good selectivity for N_2_ formation, and excellent thermal stability in the harsh conditions of exhaust after-treatment systems.^1,2^

The NH_3_-SCR reaction is a redox reaction following the equation 4NO + 4NH_3_ + O_2_ → 4N_2_ + 6H_2_O. The NH_3_-SCR activity of Cu-CHA catalysts is due to the capability of the Cu ions to reversibly change the oxidation state between Cu^I^ and Cu^II^.^3−5^ In the NH_3_-SCR reaction cycle, Cu^II^ is reduced to Cu^I^, followed by a reoxidation of the Cu^I^ to restore the Cu^II^. The reaction cycle can be performed stepwise, by alternating a reduction in a mixture of NO and NH_3_, and an oxidation in a mixture of NO and O_2_.^4,6−9^ For the reduction half-cycle, there is converging evidence that the reduction of Cu^II^ by NO and NH_3_ at around 200 °C results in the formation of linear [Cu^I^(NH_3_)_2_]^+^ complexes. These complexes are weakly bound to the zeolite, and therefore mobile.^3,6,10,11^

In the oxidation half cycle, the Cu^I^ species reacts with O_2_ to form a Cu^II^ species, and at low temperatures, O_2_ exclusively reacts with the Cu^I^ species. Therefore, the reaction of O_2_ with the linear [Cu^I^(NH_3_)_2_]^+^ complexes is an essential step in the NH_3_-SCR reaction cycle at low temperature.^12^ To complete the activation and dissociation of the O_2_ molecule, four electrons are required. As a single Cu^I^ is capable of delivering only one electron (no evidence for Cu^III^ formation has ever been reported in the numerous studies on NH_3_-SCR), this means that other electron sources are required, which can be other Cu^I^ ions, NO or other reaction intermediates.^4−6,13,14^ Following these thoughts, it has been shown that the dissociation of O_2_ becomes easier when a single O_2_ molecule interacts with two Cu^I^ ions simultaneously to form Cu-pairs.^3,4,7,15,16^ Combining this with the mobility of the linear [Cu^I^(NH_3_)_2_]^+^ complexes, a reaction mechanism has been worked out where Cu-pair formation is facilitated by diffusion of the [Cu^I^(NH_3_)_2_]^+^ complexes inside the zeolite.^4,13^ Such a mechanism involving the formation of Cu-pairs is supported by the observation that the NH_3_-SCR rate at low temperature is, for low Cu contents, proportional to the square of the Cu content in the catalyst.^4,12,17,18^ At higher temperatures, the [Cu^I^(NH_3_)_2_]^+^ complexes decompose,^10,19^ and the Cu^I^ is then expected to lose its mobility. As a result, the formation of Cu-pairs, and therefore also the activation of O_2_, becomes more difficult. This seems to be the reason for the often observed decrease in NO_*x*_ conversion with increasing temperatures around 300 °C, which separates the low- and high-temperature regimes for Cu-CHA catalysts.^4^ At high temperatures, the reaction may occur on isolated ZCu^I^ sites (where Z indicates coordination to zeolite oxygens in the proximity of an Al exchange site), possibly mediated by the formation of Cu-nitrate and Cu-nitrite species,^2,6,15,16,20^ which then further reacts with ammonia to yield N_2_ and H_2_O.

In a model where the formation of Cu-pairs is facilitated by diffusion of [Cu^I^(NH_3_)_2_]^+^ complexes, the actual active center for the activation O_2_ is not directly associated with a specific site or location of the Cu in the zeolite. In a fresh Cu-CHA material, the positive Cu ions balance the negative charges in the zeolite framework induced by the Al substitution; a Cu^II^ ion is anchored by either a single framework Al atom, as a Z[Cu^II^(OH)] species, or by two framework Al atoms, to yield a Z_2_Cu^II^ species. These Cu species are then located either in a double 6-membered ring or an 8-membered ring. Upon exposure to a reaction gas for NH_3_-SCR, which contains NO, NH_3_, O_2_ and H_2_O, these Z[Cu^II^(OH)] and Z_2_Cu^II^ species become solvated by NH_3_ leading to the formation of the mobile [Cu^I^(NH_3_)_2_]^+^ complexes. These complexes are able to diffuse to about 9 Å away from their anchor point in the time scale of catalytic turnover, which enables the Cu-pair formation necessary for the activation of O_2_.^4,17^ This means that the original location of the Cu ions does not immediately affect the reactivity of the Cu, but the different local environments of the Cu ions may affect the solvation of the Cu by ammonia. Furthermore, Density Functional Theory (DFT) calculations have shown that the formation of Cu pairs from two [Cu^I^(NH_3_)_2_]^+^ complexes becomes more difficult in areas with two Al atoms located close to each other.^21^

**Figure 1:**
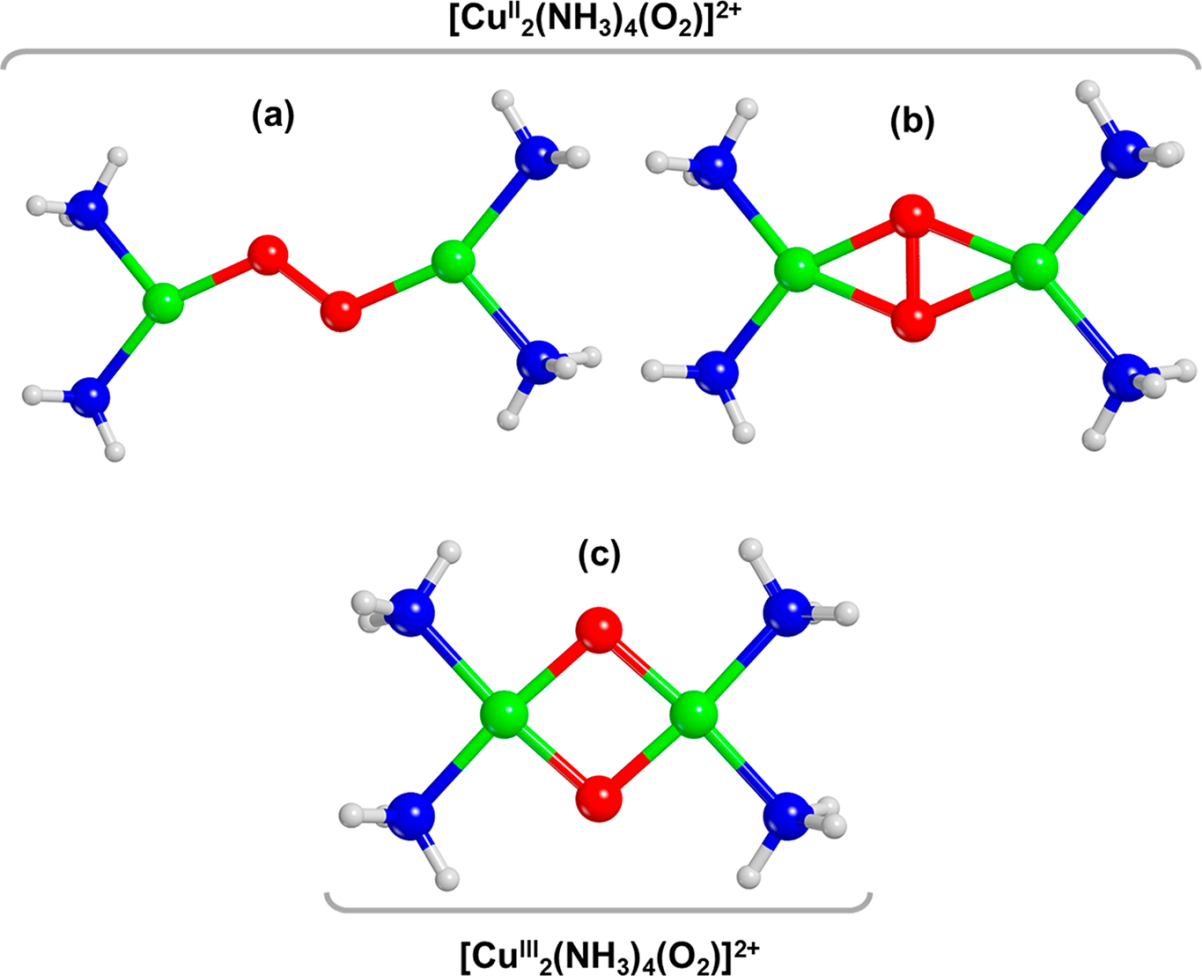
Pictorial representation of the [Cu_2_(NH_3_)_4_O_2_]^2+^ complexes proposed in ref (17): (a) *trans*-μ-1,2-peroxo diamino dicopper(II) (end-on), (b) μ-η^2^,η^2^-peroxo diamino dicopper(II) (side-on), and c) bis-μ-oxo diamino dicopper(III). Atom color code: Cu, green; H, white; O, red; N, blue.

The structures that are formed after the reaction of an O_2_ molecule and two [Cu^I^(NH_3_)_2_]^+^ complexes are Cu complexes containing two Cu-centers bridged by oxygen; the general formula of these complexes is [Cu_2_(NH_3_)_4_O_2_]^2+^. Efforts to determine the structure of this complex have mainly been based on DFT calculations. Figure 1 shows three possible structures of the [Cu_2_(NH_3_)_4_O_2_]^2+^ complexes, which differ in the way the oxygen molecule is bound to the Cu, and whether dissociation of the O–O bond takes place or not. The stability of these structures calculated with DFT depends on the functional chosen in the calculation.^22^ Calculations on ammonia-ligated Cu_2_O_2_ cores in the gas phase have shown how the predicted stability of different structures depends on the method (DFT vs post-Hartree–Fock), due to the differences in the description of the electron correlation contribution to the core conformation.^23,24^ More recent DFT calculations on O_2_ activation and dissociation by [Cu^I^(NH_3_)_2_]^+^ complexes in CHA also showed a strong dependence of the calculated stabilities and structural parameters on the selected functional. DFT calculations using a PBE functional, with or without van der Waals corrections, often result in the bis-μ-oxo diamino dicopper(III) complex^17,22^ (Figure 1c), which implies that the reaction of O_2_ with [Cu^I^(NH_3_)_2_]^+^ complexes results in the dissociation of the O–O bond. With a HSE06 hybrid functional, one finds a μ-η^2^,η^2^-peroxo diamino dicopper(II) complex (Figure 1b), in which the O_2_ molecule binds with the Cu-centers in a side-on configuration without dissociation of the O–O bond.^22^ Including a Hubbard-U term of 6 eV in DFT (DFT+U) with a PBE functional, and van der Waals corrections results in a correct prediction of the dissociation of the O–O bond in Cu_2_O_2_ complexes in enzymes with a structure similar to those expected in the Cu-CHA zeolite.^22^ Calculations of the [Cu_2_(NH_3_)_4_O_2_]^2+^ complex with that method pointed to the formation of the μ-η^2^,η^2^-peroxo diamino dicopper(II) complex (Figure 1b),^22^ just like the HSE06 functional. Clearly, experimental data for the structure of the [Cu_2_(NH_3_)_4_O_2_]^2+^ complex are needed to resolve this issue.

In this work, we present a spectroscopic investigation of the structure and reactivity of the [Cu_2_(NH_3_)_4_O_2_]^2+^ complexes formed upon reaction of O_2_ with the mobile [Cu^I^(NH_3_)_2_]^+^ complexes in a Cu-CHA catalyst for NH_3_-SCR. The available X-ray absorption spectroscopy data for Cu-CHA at the end of the transient O_2_ oxidation experiment are not conclusive on the detailed structure of the [Cu_2_(NH_3_)_4_O_2_]^2+^ complexes.^17^ The concept that dissociation of O_2_ requires two solvated Cu^I^ ions also opens the question whether Cu pairs play a role beyond O_2_ activation. Therefore, we also investigate the reactivity of the [Cu_2_(NH_3_)_4_O_2_]^2+^ complexes toward NO and NH_3_. These goals have been pursued by applying X-ray absorption spectroscopy (XAS), both near-edge (XANES) and extended range (EXAFS), with a well-established *operando* setup,^6,10,25^ and diffuse reflectance ultraviolet–visible–near-infrared (DR UV-Vis-NIR) spectroscopy^19,26^ using a Cu-CHA catalyst. DFT calculations were used for optimization of the [Cu_2_(NH_3_)_4_O_2_]^2+^ structures illustrated in Figure 1, which were used as input for the interpretation of the EXAFS data. To enhance the sensitivity of EXAFS to multicopper moieties, we applied wavelet transform (WT) analysis, giving unprecedented insights in the formation and separation of the solvated Cu-pairs in the NH_3_-SCR reaction cycle over Cu-CHA catalysts.

## 2 Experimental Section

**Scheme 1:**
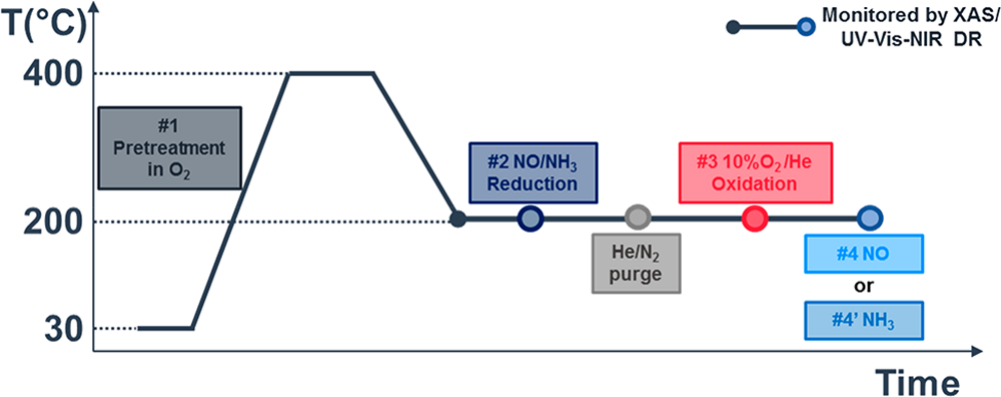
Experimental Steps Followed by XAS and UV-Vis-NIR DR Spectroscopies Step 1, pretreatment at 400 °C in pure O_2_; step 2, reduction in 1000 ppm of NO/1000 ppm of NH_3_/He; step 3, oxidation in 10% O_2_/He; step 4, reaction with 1000 ppm of NH_3_/He or step 4′, with 1000 ppm of NO/He. Total gas flow: 100 mL/min for XAS, 50 mL/min for UV-Vis. The purge step was carried out in He for XAS and in N_2_ for UV-Vis.

The catalyst used in this study was a Cu-CHA material with Si/Al = 15 with a Cu content of 2.6 wt%, corresponding to Cu/Al = 0.5, a Cu density of around 0.4 Cu/1000 Å^3^ (0.3 Cu per chabazite cage) and a theoretical mean Cu–Cu distance of 16.6 Å. At this Cu density, about 90% of Cu^I^ ions can be oxidized by O_2_, forming the [Cu_2_(NH_3_)_4_O_2_]^2+^ complexes.^17^ The steps depicted in Scheme 1 have been used to form these complexes and to study the reactivity toward NO and NH_3_ experimentally. These steps are as follow:

1. pretreatment in O_2_ at 400 °C;
2. reduction in a mixture of 1000 ppm of NO and 1000 ppm of NH_3_ at 200 °C to form [Cu^I^(NH_3_)_2_]^+^;^4,6,17,27^
3. oxidation of the [Cu^I^(NH_3_)_2_]^+^ complexes by O_2_ at 200 °C to form the [Cu_2_(NH_3_)_4_O_2_]^2+^ complexes;^17^
4. reaction of the [Cu_2_(NH_3_)_4_O_2_]^2+^ complexes with NO or NH_3_ at 200 °C

The steps in this protocol were followed independently by XAS coupled to an online mass spectrometer for a qualitative effluent gas analysis (BM23 beamline of the European Synchrotron Radiation Facility),^28^ and by DR UV-Vis-NIR. In this Article, we present the development of spectra with time under isothermal conditions, until a steady-state was observed. The DR UV-Vis-NIR spectra are reported as relative reflectance (R%), to avoid artifacts due to the use of the Kubelka–Munk function.^26^ We refer to the Supporting Information (SI), section 1, for more experimental details and description of the equipment used.

## 3 Results

### 3.1 Oxidation of [Cu^I^(NH_3_)_2_]^+^ Complexes

#### 3.1.1 Oxidation and Coordination State of Cu Ions

**Figure 2:**
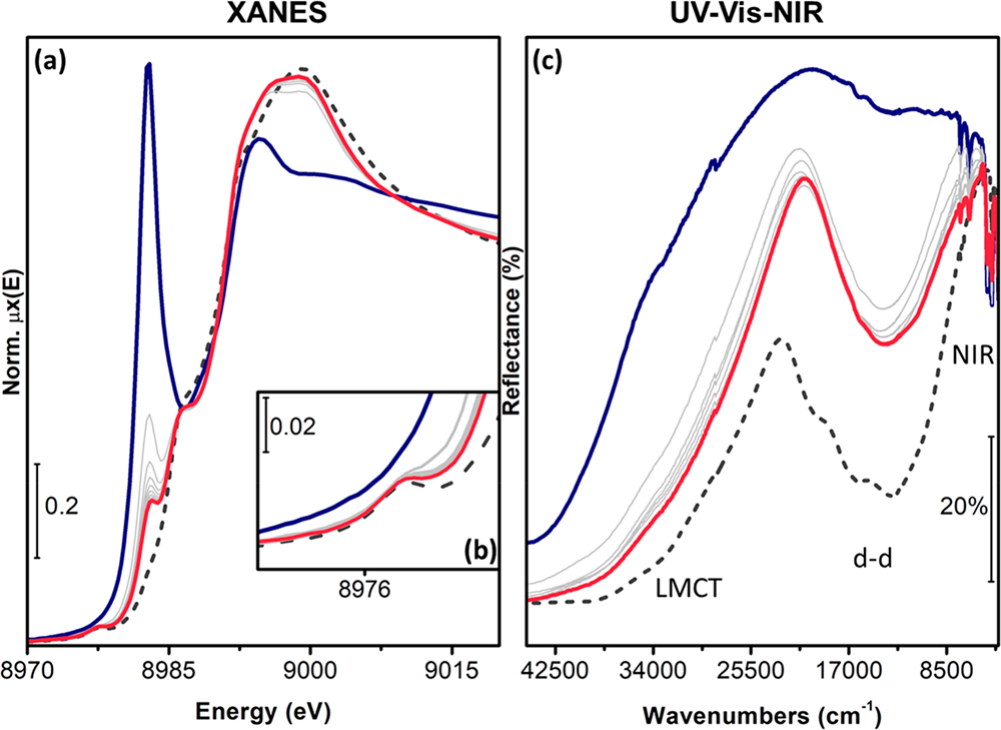
Evolution of the Cu K-edge XANES (a) and DR UV-Vis-NIR spectra (c) of Cu-CHA during exposure to 10% O_2_/He at 200 °C after reduction in NO/NH_3_/He at 200 °C. Inset (b) reports a magnification of the pre-edge peak arising from the Cu^II^ 1s →3d transition. Dark blue thick line: after NO/NH_3_/He exposure; red thick line: final spectrum after exposure to 10% O_2_; gray thin lines: intermediates; dark gray dashed line: Cu-CHA pretreated in O_2_, by heating in O_2_ up to 400 °C and subsequently cooling in O_2_ down to 200 °C prior to XAS data collection, step 1.

The linear [Cu^I^(NH_3_)_2_]^+^ complexes are the starting point of the experiment discussed in this section. These complexes are formed by exposure of the pretreated Cu-CHA catalyst (step 1) to a NO/NH_3_ mixture at 200 °C (step 2). The presence of this state is indicated by a characteristic rising-edge peak at ∼8982.5 eV in the Cu K-edge XANES spectrum, corresponding to the 1s→4p transition of linearly coordinated Cu^I^ centers (Figure 2a, blue curve) and validated by EXAFS fitting based on the [Cu^I^(NH_3_)_2_]^+^ model (see SI, section 2.2). Upon reaction of this linear [Cu^I^(NH_3_)_2_]^+^ complex with a mixture of 10% O_2_ in He, this distinct peak at 8982.5 eV disappears almost completely, the XANES resembles that observed after pretreatment in O_2_ (Figure 2a, red and gray dashed lines) and the dipole-forbidden 1s→3d transition of Cu^II^ becomes visible at 8977.3 eV (Figure 2b). These changes clearly indicate that the linear [Cu^I^(NH_3_)_2_]^+^ complex is oxidized by O_2_. After 30 min in O_2_/He, a small feature at 8982.5 eV is still recognized, but the spectra do not change any more. This indicates, that not all [Cu^I^(NH_3_)_2_]^+^ complexes are oxidized; in agreement with earlier observations^17^ about 10–20% of the [Cu^I^(NH_3_)_2_]^+^ complexes do not react, based on qualitative XANES analysis. The mass spectrometer connected to the cell did not detect any NH_3_ desorption during the oxidation, indicating that the Cu does not lose the NH_3_ ligands in the oxidation process.

This evidence points to the presence of different, NH_3_-solvated Cu^II^ species with respect to the framework-coordinated Cu^II^ ions (fw-Cu^II^) known to be present in the catalyst after pretreatment in O_2_.^7,25,29−31^ To strengthen such indications, we initially analyzed by linear combination fit (LCF) the XANES after oxidation in O_2_ (step 3) using the spectra obtained at step 1 (pretreated in O_2_) and at step 2 (reduced in NO/NH_3_) as references for Cu^II^ and Cu^I^ components, respectively (see SI, Figure S11). Overall, estimates for Cu^I^ and Cu^II^ percentages are in reasonable agreement with qualitative analysis. It is also evident that the rising edge peak at 8982.5 eV diagnostic of Cu^I^ species is excellently reproduced, indicating that the Cu^I^ component, i.e., linear [Cu^I^(NH_3_)_2_]^+^, is the same at both steps 2 and 3. However, significant discrepancies between experimental and LCF curve are found when considering the rising-edge region where Cu^II^ 1s→4p transitions typically occurs, as well as the shape and energy position of the white-line peak. These discrepancies translate into a structured residual function, with well-defined maxima and minima well above the noise level, further indicating that two spectroscopically distinguishable Cu^II^ species are present at steps 1 and 3.

In the UV-Vis-NIR spectra, the oxidation of the linear [Cu^I^(NH_3_)_2_]^+^ complex is visible as follows. Due to the d^10^ closed-shell configuration of Cu^I^, the typical ligand-field d-d transitions are absent, and the spectrum is dominated by a ligand-to-metal charge transfer (LMCT) transition observed in the range 30 000–45 000 cm^–1^ (blue curve in Figure 2c).^26,32^ The oxidation of Cu^I^ to Cu^II^ by O_2_ is reflected in the development of an intense d-d absorption centered at 13 850 cm^–1^ and a red-shift of the LMCT transitions, from ca. 35 000 to 25 000 cm^–1^ (arbitrarily measured at *R* = 60%). The features at 6515 and 4970 cm^–1^ in the NIR region are due to the overtones and combination modes of NH_3_ and NH_4_^+^, confirming that the Cu^II^ species after oxidation still contains the NH_3_ ligands. The spectrum of the Cu-CHA catalyst pretreated in O_2_ (dark gray dashed curve) is reported for comparison, showing that the coordination geometry of the Cu^II^ species formed by oxidation of [Cu^I^(NH_3_)_2_]^+^ is different with respect to that of the variety of framework-coordinated monomeric/multimeric ions (fw-Cu^II^, such as Z[Cu^II^(OH)]/Z[Cu^II^(OO*)] etc.) responsible for the typical Cu-CHA “quadruplet” (complex absorption in the d-d region with components at 20 000, 16 350, 13 300, and 10 600 (sh) cm^–1^).^26,33−36^

#### 3.1.2 Structure of the [Cu_2_(NH_3_)_4_O_2_]^2+^ Complexes

**Figure 3:**
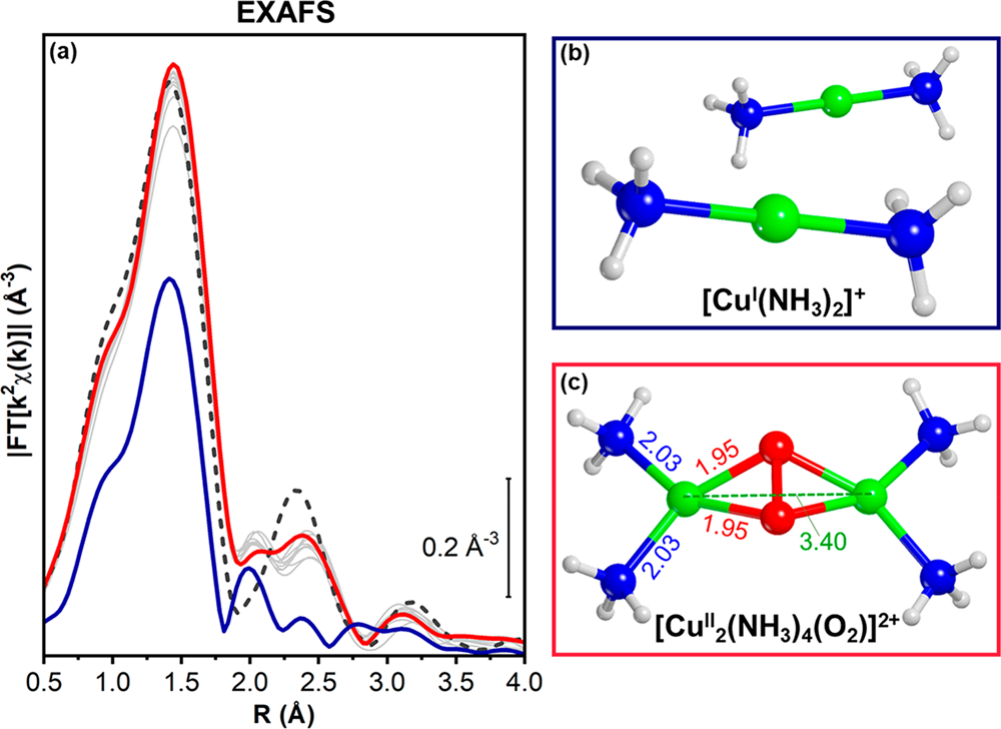
(a) Phase-uncorrected *k*^2^-weighted FT-EXAFS curves during exposure of the Cu-CHA catalyst to NO/NH_3_/He (dark blue thick line), followed by 10% O_2_ in He (gray thin lines; red thick line: final spectrum; dark gray dashed line: pretreatment in O_2_ by heating in O_2_ up to 400 °C and subsequently cooling in O_2_ down to 200 °C prior to XAS data collection, step 1. (b) Illustration of [Cu(NH_3_)_2_]^+^ and (c) μ-η^2^,η^2^-peroxo diamino dicopper(II) (side-on) complexes. Atom color code: Cu, green; H, white; O, red; N, blue. Part (c) also report selected DFT bond distances in Å.

To determine the precise structure of the Cu^II^ complex formed by the oxidation of the linear [Cu^I^(NH_3_)_2_]^+^ species with O_2_, we have analyzed the Fourier-transformed (FT) EXAFS spectra; Figure 3a shows these data without phase correction. The first-shell peak is almost doubled in intensity after O_2_ interaction (from blue to red), indicating that the coordination number of Cu in the Cu^II^ complexes is higher than in the linear [Cu^I^(NH_3_)_2_]^+^ complex. In the next shell, the unstructured feature observed for the mobile [Cu^I^(NH_3_)_2_]^+^ complex evolves toward a broad scattering feature peaking at ca. 2.4 Å. Even though this feature is close to the second-shell EXAFS signature of framework-coordinated Cu^II^ ions after pretreatment in O_2_ (dashed gray line), it can be clearly distinguished, indicating that, at least a substantial part of the Cu^II^ complex is still mobile in the cage. Finally, a third peak around 3.2 Å develops, which could correspond to contributions from a Cu–Cu scattering in the Cu^II^ complexes shown in Figure 1. All these features in the FT-EXAFS are consistent with the formation of [Cu_2_(NH_3_)_4_O_2_]^2+^ complexes. However, a more detailed analysis is required to rule out re-coordination of Cu^II^ ions to zeolite framework and, once this possibility is excluded, to identify the precise [Cu_2_(NH_3_)_4_O_2_]^2+^ structure, based on the three possibilities shown in Figure 1.

To this aim, we have first performed quantitative EXAFS analysis of the spectra obtained at step 1 (gray dashed line in Figure 3). The adopted fitting model is based on structural characteristics conserved for most fw-Cu^II^ species previously proposed to form in Cu-CHA upon pretreatment in O_2_.^7,25,29−31,34^ Importantly, it accounts for a distinctive scattering contribution of charge-balancing Al atoms (Al_fw_) located at ca. 2.75 Å range from the Cu^II^ center, while maintaining a certain degree of flexibility to account for fractional contribution from multicopper species (additional information can be found in SI, section 2.1). As detailed in the SI, the fit resulted in a good level of reproduction of experimental EXAFS spectrum for the pretreated catalyst at step 1, with physically meaningful values for all the refined parameters, as well as for their fitting errors (see SI, Figure S2 and Table S2).

A second stage of our analysis consisted in a test EXAFS fit of the experimental spectrum of the [Cu(NH_3_)_2_]^+^ complexes oxidized in O_2_ (step 3, red solid line in Figure 3), using exactly the same model based on fw-Cu^II^, which guaranteed a successful fit for the EXAFS of the pretreated catalyst (see SI, section 2.3). While the numerical agreement between best-fit and experimental curve was formally satisfactory, this was achieved at the expense of the physical meaning of the optimized parameters (e.g., Debye–Waller factors as high as 0.1 Å^2^) as well as of their accuracy (unphysically high fitting errors). Such inconsistencies most severely affect the Cu–Al_fw_ coordination shell, representing a diagnostic contribution for the large majority of fw-coordinated Cu^II^ species proposed so far in the CHA framework. Consistently with the LCF XANES results in SI, Figure S11, the failure of this test EXAFS fit strongly supports the spectroscopically detectable diversity of Cu^II^ species formed in Cu-CHA at steps 1 and 3. The state of the catalyst at step 3 is clearly not consistent with fw-coordinated Cu^II^ species, thus paving the way to deeper structural analysis considering mobile [Cu^II^_2_(NH_3_)_4_O_2_]^2+^ complexes.

To determine the precise structure of the [Cu_2_(NH_3_)_4_O_2_]^2+^ complexes, first we have optimized the structures in the gas phase (that is not including the zeolite effect in the calculations) for the three complexes shown in Figure 1 by DFT, including the three main structures proposed in the literature by different authors: NH_3_-solvated side-on^22^ and end-on^4^ peroxides as well as the bis(μ-oxo)-dicopper core.^17^ Then, these structures were used as input to fit the observed EXAFS features. A detailed description of the DFT and fitting procedures and results can be found in the SI, section 2.4. Note that, due to the difficulty in determining the relative stabilities of the [Cu_2_(NH_3_)_4_O_2_]^2+^ complexes by DFT,^22−24^ we have only used the DFT calculations to provide reasonable structures for the [Cu_2_(NH_3_)_4_O_2_]^2+^ complexes as input for fitting the EXAFS data.

The difference in the structures of the [Cu_2_(NH_3_)_4_O_2_]^2+^ complexes in Figure 1 lays in the Cu_2_O_2_ cores. The Cu_2_O_2_ cores differ in the Cu–Cu, Cu–O, and O–O bond lengths, and in the oxidation state of the Cu. Table 1 contains a comparison of our calculated values for the different possible [Cu_2_(NH_3_)_4_O_2_]^2+^ complexes and the known values for similar Cu_2_O_2_ cores from enzymes, where the precise structure is determined experimentally.^37−41^ From the table, we observe that our calculated values agree well with the experimentally determined values for enzymes, which validates our structural models derived from DFT calculations for further EXAFS analysis.

**Table 1:** Experimental and Calculated Structural Parameters of Cu_2_O_2_ Cores

	bond length (Å)				
	Cu–Cu	Cu–O	O–O	Cu oxidation state	ref
*trans* (end-on) μ-1,2-peroxo dicopper(II)	4.36	–	1.43	2+	(38,41)
	4.149–4.300	1.860–2.113	1.270–1.449		This work^a^

(side-on) μ-η^2^,η^2^-peroxo dicopper(II)	3.52–3.59	1.97–1.98	1.37–1.54	2+	(38,39,42)
	3.059–3.597	1.910–1.983	1.416–1.508		this work^a^

bis-μ-oxo dicopper(III)	2.74–2.83	1.80–1.83	2.29–2.37	3+	(37−39,42)
	2.644–2.872	1.772–1.870	2.265–2.359		this work^a^

EXAFS best fit	3.40 ± 0.05	1.911 ± 0.009	–	–	this work

a DFT calculations on zeolite-free molecular complexes with M06-HF-D and M06-L-D functionals.

Fitting the EXAFS data with the (end-on) trans μ-1,2-peroxo diamino dicopper(II) or bis-μ-oxo diamino dicopper(III) models (Figure 1a,c) lead unsatisfactory results (see SI, section 2.4), and therefore, the observed EXAFS features are not consistent with these structures. Using the side-on μ-η^2^,η^2^-peroxo diamino dicopper(II) complex (Figure 1b and Figure 3c) to fit the EXAFS data, we find a Cu–O distance of 1.911 ± 0.009 Å, a Cu–N distance of 2.06 ± 0.02 Å, and a Cu–Cu distance of 3.40 ± 0.05 Å. These results are in excellent agreement with values calculated by DFT, and also match the values for a similar Cu_2_O_2_ core in enzymes (Figure 3c and Table 1). Furthermore, the EXAFS fit based on the μ-η^2^,η^2^-peroxo diamino dicopper(II) complex also revealed intense multiple scattering contributions in the 2.5–3.5 Å range—mostly triangular scattering paths involving the two O atoms of the peroxo group and quasi-collinear paths across the N(NH_3_)–O(peroxo) diagonal. This points to the fact that the majority of the formed [Cu_2_(NH_3_)_4_O_2_]^2+^ complexes are characterized by the μ-η^2^,η^2^-peroxo diamino dicopper(II) structure depicted in Figure 3c.

Having identified the [Cu_2_(NH_3_)_4_(O_2_)]^2+^ complex as μ-η^2^,η^2^-peroxo diamino dicopper(II), we further refined the individual contributions of this complex and the unreacted [Cu^I^(NH_3_)_2_]^+^ complexes by multi-component EXAFS fitting (see SI, section 2.4.3 for details on the procedure). We find that 16 ± 8% of Cu is still present as [Cu^I^(NH_3_)_2_]^+^, indicating that not all [Cu^I^(NH_3_)_2_]^+^ are oxidized by O_2_. The amount of unreacted [Cu^I^(NH_3_)_2_]^+^ complexes we find here is in good agreement with the estimate based on the qualitative XANES analysis presented above, and with theoretical predictions based on a limited mobility of the [Cu^I^(NH_3_)_2_]^+^ complexes at the studied Cu density.^17^

#### 3.1.3 Validating the Structural Dynamics of Cu Species by EXAFS Wavelet Transform (WT) Analysis

The DFT-guided EXAFS fitting results allow us to define a consistent experimentally based model for the [Cu_2_(NH_3_)_4_(O_2_)]^2+^ complexes previously proposed.^4,17^ A conclusive assignment of the features observed in conventional Fourier transform (FT) EXAFS spectra, however, is hindered by the simultaneous presence of various types of atomic neighbors surrounding the Cu absorber, especially in the high-*R* region. If two or more types of elemental neighbors and/or scattering interactions are localized at close distances around the absorber, their contributions in the direct space *R* overlap and often become indistinguishable. For the Cu-CHA zeolite studied here, these potentially include single scattering paths from framework Al/Si/O in zeolite-coordinated Cu moieties as well as Cu in multicopper species, which can be coordinated to the zeolite or mobile.^29^ The intense multiple scattering paths involving first-shell O/N neighbors in the proposed μ-η^2^,η^2^-peroxo diamino dicopper(II) moiety mentioned above are also expected to fall in this *R*-space range.

**Figure 4:**
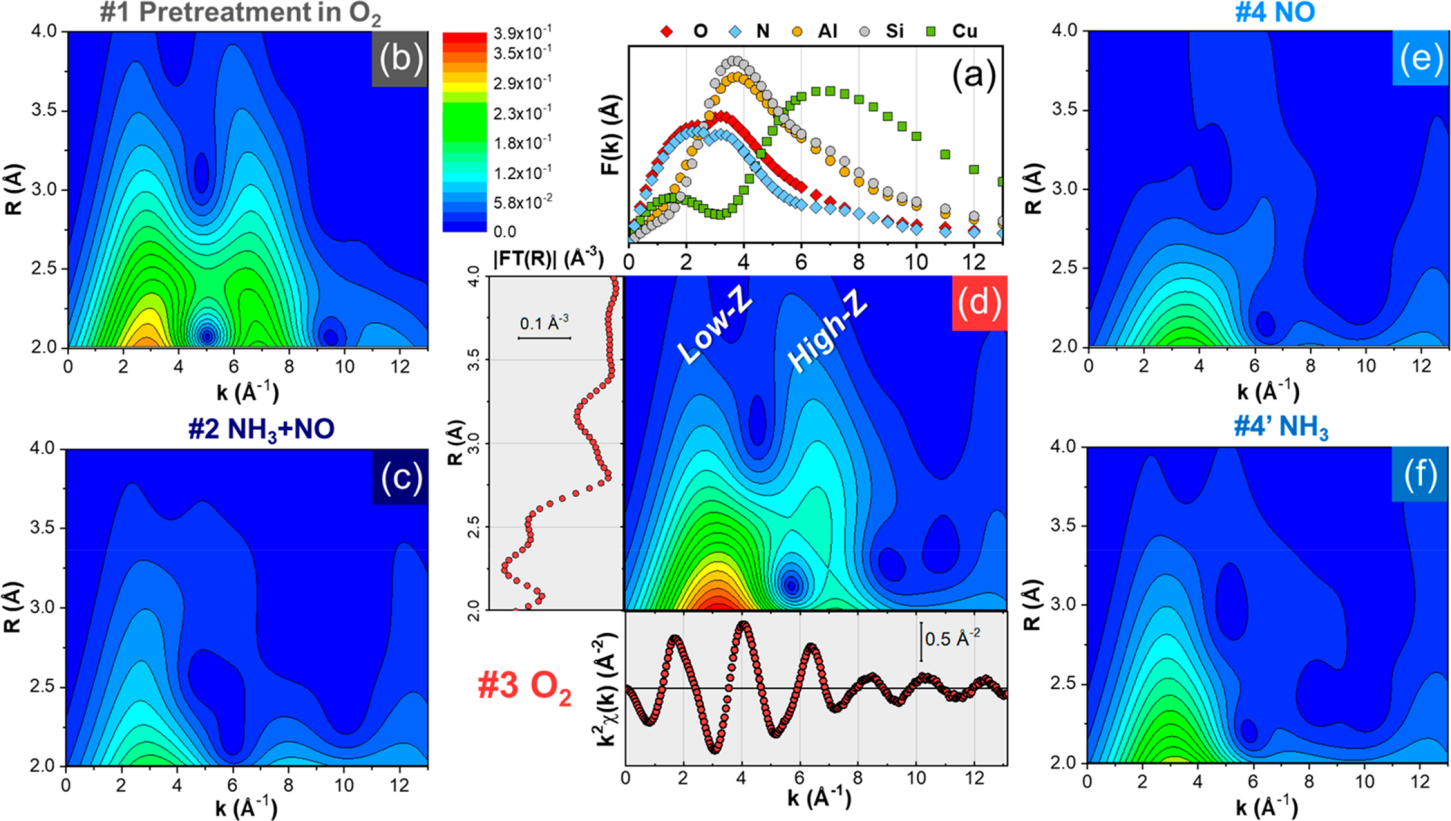
(a) Backscattering amplitude factors associated with the elements present in the system under study. Moduli of EXAFS WTs magnified in the 2–4 Å *R*-space range for the following reaction steps: (b) step 1, pretreatment in O_2_; (c) step 2, reduction in NO/NH_3_/He; (d) step 3, oxidation in 10% O_2_/He; (e) step 4, exposure to NO/He or (f) step 4′, exposure to NH_3_/He. For all the WTs a common intensity scale is employed. Part (d) also shows the corresponding EXAFS spectra in *k*-space and *R*-space (conventional FT) in the relevant ranges, as well as the *k*-space range characteristic of low-*Z* (O/N, Si/Al) and high-*Z* scatterers (Cu). All spectra measured at 200 °C.

To resolve this, it is possible to exploit the fact that the contributions from different elemental neighbors appear at different locations in *k*-space, because the backscattering amplitude factor *F*(*k*) strongly depends on the atomic number *Z*. Figure 4a shows the *F*(*k*) curves associated with the elements relevant in this work, namely O, N, Al, Si, and Cu. It is clear that signals produced by heavier atoms, such as Cu, are localized at higher *k* values with respect to lighter atoms. On this background, a WT analysis allows for a better discrimination of the nature of the scattering contributions around the absorber, compared to the classical FT analysis.^30,43−47^ The WT analysis results in a 2D representation of the EXAFS, simultaneously revealing the signal features in both *R*- and *k*-space. Then, one can visually resolve the scattering contributions originating from atomic neighbors having enough *Z*-contrast in their *F*(*k*) functions. A more detailed description of the WT analysis technique is given in the SI, section 3.1.

Figure 4b–f reports the moduli of EXAFS WTs for the key reaction steps explored in this work, measured in steady-state conditions at 200 °C. Here, EXAFS WTs are magnified in the 2–4 Å *R*-space range, where signal interpretation with conventional FT-EXAFS approach is mostly complicated by overlapping contributions. Corresponding full-range EXAFS-WTs are reported and discussed in detail in the SI, section 3.3.

In the relevant *R*-space range, EXAFS WT for the spectrum collected in O_2_ at 200 °C after pretreatment in O_2_ at 400 °C (Figure 4b), clearly splits in two lobes. The first sub-lobe, localized in the *k* range 1–5 Å^–1^ and *R* range 2–2.8 Å is associated with the framework atoms: O, Si and Al. The second one, localized at higher *k* values (i.e., 6–8 Å^–1^), is principally related to Cu–Cu contributions in oxygen-bridged Cu dimers or, more in general, multicopper moieties. WT analysis further validate the previously mentioned EXAFS fitting results for step 1 based on a prototypical fw-Cu^II^ model (see SI, section 2.1), confirming the simultaneous presence of Cu–Al, Cu–O/Si, and Cu–Cu scattering contributions in the high-*R* EXAFS range for the pretreated catalyst. The presence of Cu-oxo dimeric/polymeric cores in oxygen activated Cu-CHA catalysts represents a novelty with respect to previous literature in the context of the NH_3_-SCR reaction.^25^ This aspect has been recently established by different authors studying the nature of Cu-oxo species in Cu-CHA for the direct methane to methanol conversion.^26,29,30,34,35,43,47^ While from the EXAFS fit in SI, section 2.1 an average Cu–Cu coordination number N_Cu_ = 0.5 ± 0.3 is estimated, a more precise identification of the nature and amount of these dimeric/polymeric structures in the oxygen activated Cu-CHA is outside the scope of this manuscript and probably beyond the possibilities of the technique. However, the contrast between *F*(*k*) function for Cu and for the rest of relevant elements in the system is sufficient to reveal the presence of (a fraction) of multicopper moieties.

Indeed, the EXAFS backscattering amplitude factors in Figure 4a show that the *F*(*k*) functions of lighter elements, such as O/N and Si/Al, have maxima at around 3–4 Å^–1^, while for Cu, the position of the main peak significantly shifts to a *k*-value of around 7 Å^–1^. These differences lead to the observed lobe splitting, enabling an unambiguous, visual discrimination of contributions stemming from Cu or framework atomic contributions. Due to the substantial overlap of the related backscattering amplitude functions, it is not possible to discriminate by means of WT among O/N and Si/Al contributions.

A WT analysis of the EXAFS spectrum for the mobile [Cu^I^(NH_3_)_2_]^+^ complexes (Figure 4c), which is obtained after exposure of the catalyst pretreated in O_2_ to the NO/NH_3_ gas mixture at 200 °C (step 2), shows a complete reduction and mobilization of the Cu ions in the system.^6,10,17,19,48^ The second-shell peak in the conventional FT-EXAFS disappears (Figure 3a and SI, Figure S6) and all the high-R features are substantially decreased in the corresponding WT map in Figure 4c. Even though the sub-lobe at *k* = 7 Å^–1^ associated with the Cu–Cu signal is completely lost at this step, a low-intensity feature is still visible in the *k*-space range 1–5 Å^–1^, which is most likely due to multiple scattering paths involving the first-shell N ligands in the linear [Cu^I^(NH_3_)_2_]^+^ moieties.

The crucial step for the activation of oxygen is the isothermal oxidation of the [Cu^I^(NH_3_)_2_]^+^ complexes, and the corresponding WT analysis is shown in Figure 4d. The most important observation here is the appearance of a sub-lobe at around *k* = 7 Å^–1^ indicating a contribution of Cu–Cu scattering. The presence of this feature unambiguously demonstrates the formation of a Cu complex containing more than one Cu atom. This agrees well with the formation of [Cu_2_(NH_3_)_4_O_2_]^2+^ complexes, and is in line with our EXAFS results (section 3.1.2) and earlier work.^4,16−18^ The second sub-lobe occurs in the same *k*-space range observed in the EXAFS WT after pretreatment in O_2_. However, we note a different morphology of the WT along the *R* direction in the two probed states, indicating that there is a difference in Cu–Cu coordination after pretreatment in O_2_ or oxidation of [Cu^I^(NH_3_)_2_]^+^ at 200 °C. After oxidation of [Cu^I^(NH_3_)_2_]^+^ (step 3, Figure 4d), the WT intensity is rather localized in *R*-space. It peaks at ca. 2.8 Å in the phase-uncorrected *R*-axis, pointing to a uniform Cu–Cu interatomic distance around 3.5 Å. This indicates, that the reaction of [Cu^I^(NH_3_)_2_]^+^ with O_2_ results in a well-defined structure, compatible with the side-on μ-η^2^,η^2^-peroxo diamino dicopper(II) complex shown in Figure 1b and Figure 3c. In contrast, after heating in O_2_ at 400 °C and subsequent cooling to 200 °C in O_2_ (step 1, Figure 4b), a broader intensity distribution in *R*-space is observed in the *k*-space region characteristic of Cu–Cu scattering, in agreement with the presence of more heterogeneous multicopper species in the pretreated Cu-CHA catalyst.^29,34,35^

Figure 4 also reports the EXAFS-WTs related to the reactivity of the formed μ-η^2^,η^2^-peroxo diamino dicopper(II) complexes with the key SCR reactants, NO (step 4, Figure 4e) and NH_3_ (step 4′, Figure 4f). In both cases, the sub-lobe at *k* = 7 Å^–1^ is clearly lost, providing direct structural evidence for the cleavage of dicopper cores upon separate exposure to NO or NH_3_ at 200 °C. A moderately intense sub-lobe is instead still visible in the low-*k* range, characteristic of low-*Z* scatterers. As argued before, this feature most likely stems from multiple scattering contributions involving N and O atoms.

Finally, to comparatively assess the presence of Cu–Cu scattering contributions throughout the investigated reaction steps, we computed the power density function Φ^*R*^ of the WT representation.^45^ This quantity was obtained integrating the square of the modulus of the WT over the *R*-range 2–4 Å, that should contain the Cu–Cu signal contribution. Φ^*R*^ is given by the following expression:
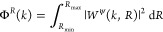
1where *R*_min_ = 2 Å, *R*_max_ = 4, and *W*^ψ^(*k*,*R*) is the wavelet transform representation of the EXAFS signal depending on the mother function used (see SI, section 3.1 for details).

**Figure 5:**
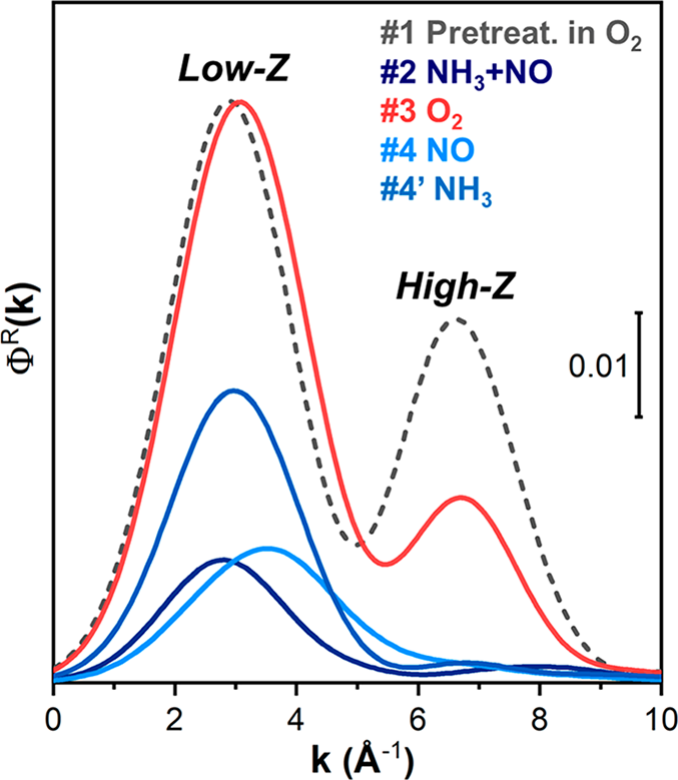
Density power function Φ^*R*^ calculated for the WT representations showed in Figure 4b–f. *k*-Space ranges diagnostic for light-*Z* scatterers (O/N, Si/Al) and high-*Z* scatterers (Cu), based on backscattering amplitude factors *F*(*k*) reported in Figure 4a, are indicated.

Figure 5 presents the results of these calculations, summarizing the above observations about EXAFS WTs. A common first peak, for all the steps, is localized in the 0.0–5.5 Å^–1^ range: it corresponds to the WT low-*k* sub-lobe, collectively accounting for the contributions from O, N, Si and Al atoms. The second main peak is present only in catalyst pretreated in O_2_ (curve 1) and after [Cu^I^(NH_3_)_2_]^+^ reaction with O_2_ (curve 3). The position of this peak exactly corresponds to the maximum of the Cu backscattering amplitude function shown in Figure 4a, clearly indicating the formation of [Cu_2_(NH_3_)_4_(O_2_)]^2+^ complexes.

### 3.2 Reactivity of [Cu_2_(NH_3_)_4_(O_2_)]^2+^ Species toward NH_3_ and NO

To determine the reactivity of μ-η^2^,η^2^-peroxo diamino dicopper(II) complexes toward NO and NH_3_, which are the main reactants in NH_3_-SCR, we have exposed them to NO and NH_3_ separately, and in a 1:1 mixture. Exposure to a mixture of NO and NH_3_ results in a complete restoration of a Cu^I^ oxidation state as [Cu^I^(NH_3_)_2_]^+^ with formation of the N_2_ product, confirming the NH_3_-SCR reaction and the reversibility of the oxidation of the [Cu^I^(NH_3_)_2_]^+^ species (see SI, Figure S12 and qualitative mass spectrometry analysis in SI, Figure S16). This observation provides experimental evidence that it is possible to close the NH_3_-SCR reaction cycle between the above-discussed and identified “homogeneous-like” μ-η^2^,η^2^-peroxo diamino dicopper(II) and [Cu(NH_3_)_2_]^+^ complexes.

#### 3.2.1 Reactivity toward NH_3_: Structural Changes

As shown by EXAFS-WT analysis above (Figure 4f), exposure of the μ-η^2^,η^2^-peroxo diamino dicopper(II) complexes to NH_3_ results in the separation of the copper centers. No significant N_2_ evolution is observed during this transformation (see SI, Figure S17). In this section, we focus on the structural changes of the resulting Cu complexes.

**Figure 6:**
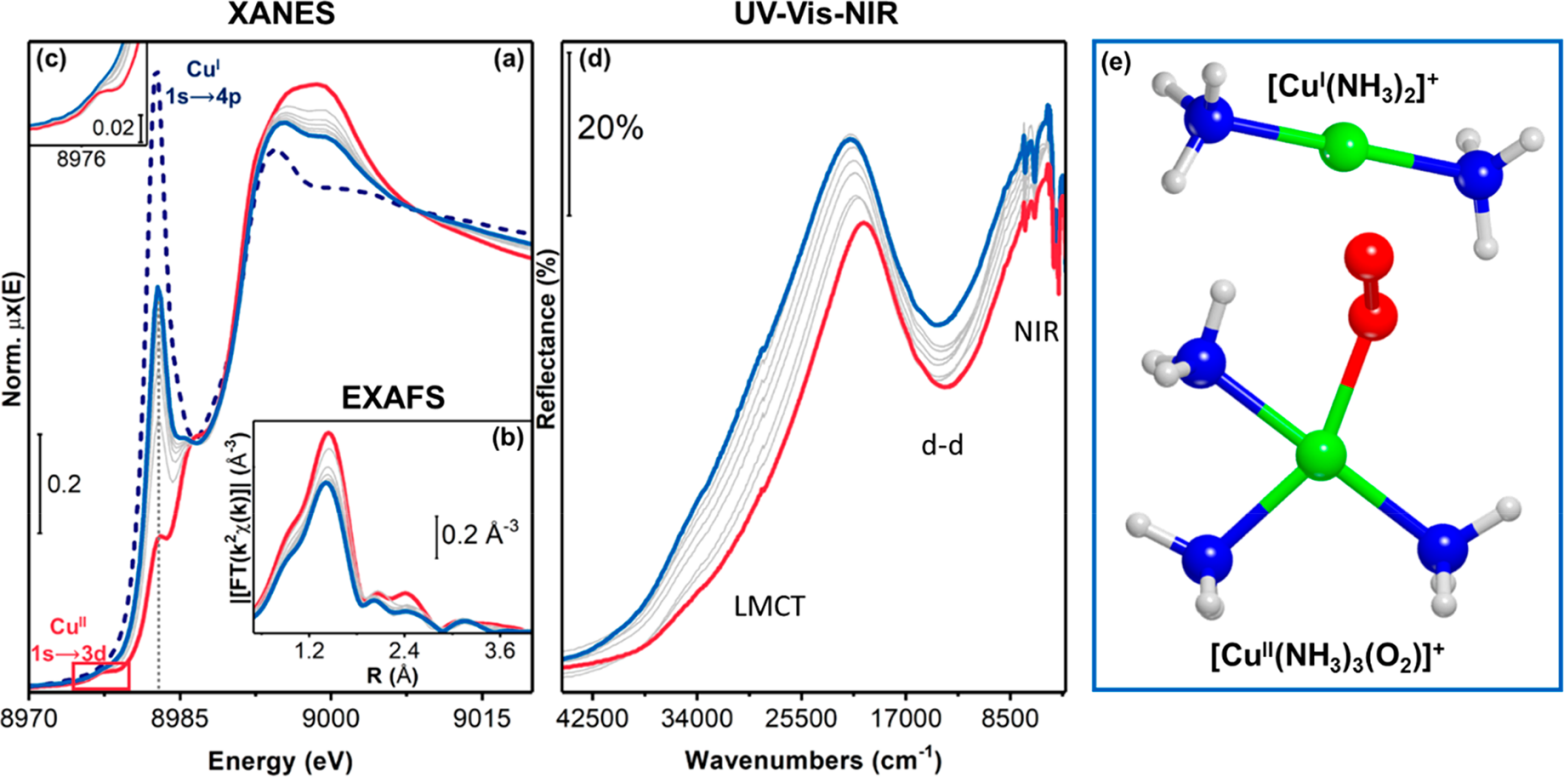
Exposure to NH_3_/He at 200 °C of Cu-CHA catalyst after step 3. (a) Cu K-edge XANES spectra. (b) Phase-uncorrected *k*^2^-weighted FT EXAFS curves. (c) Magnification of Cu^II^ 1s →3d transition. (d) UV-Vis-NIR DR spectra. Red thick line: spectrum collected in step 3 (same as Figures 2 and 3); blue thick line: after NH_3_/He exposure; gray thin lines: intermediates; dashed dark blue line : step 2 (NO/NH_3_ at 200 °C after pretreatment in O_2_, same as dark blue in Figures 2 and 3). (e) Illustration of Cu species proposed to be formed upon reaction of the μ-η^2^,η^2^-peroxo diamino dicopper(II) complexes with NH_3_. Atom color code: Cu, green; H, white; O, red; N, blue.

Figure 6 reports the XAS and UV-Vis-NIR spectra for the μ-η^2^,η^2^-peroxo diamino dicopper(II) complexes during exposure to NH_3_ at 200 °C. The characteristic Cu^I^ transition at ∼8982.5 eV for the linear [Cu^I^(NH_3_)_2_]^+^ appears, but does not reach the intensity observed in the fully reduced catalyst (dashed dark-blue curve in Figure 6a). The intensity of the 1s→3d pre-edge peak (Figure 6c) decreases, corroborating that some reduction of the Cu takes place. In the corresponding UV-Vis-NIR spectra, the intense d-d transition, which appears at around 14 000 cm^–1^ (Figure 6d) becomes less intense and shifts from 13 800 to 14 400 cm^–1^, without substantial change in the peak shape. The fact that this peak does not disappear completely, indicates that a part of the Cu remains in the Cu^II^ state. The shift is in agreement with a change in the ligands bonding the Cu^II^ ions, affecting the d-d orbital splitting.^19,32,34,49^ This is also testified by the progressive consumption of the LMCT absorption between 27 000 and 31 000 cm^–1^, which is consistent with the disappearance of the peroxo group in the diamino dicopper(II) complex.^26,34,35^ Thus, exposure of the μ-η^2^,η^2^-peroxo diamino dicopper(II) complexes to NH_3_ leads to a change in the ligands of the Cu and to a partial reduction to a Cu^I^ species.

The UV-Vis features described above only indicate the changes in the coordination sphere of Cu^II^ ions, Cu^I^ ions being essentially silent. On the other hand, XANES and EXAFS give average information on all copper species formed in this reaction step. A decrease in the average number of ligands surrounding Cu ions is indicated by the reduced intensity of the EXAFS first shell peak (Figure 6b). The intensity, shape and energy position of the XANES features suggest the presence of a mixture of [Cu^I^(NH_3_)_2_]^+^ and [Cu^II^(NH_3_)_3_(X)]^+^ complexes, with a geometry similar to the [Cu^II^(NH_3_)_3_(OH)]^2+^ species, as reported earlier.^19,32^ A XANES linear combination fit (see SI, Figure S13) indicates that the fractions of [Cu^I^(NH_3_)_2_]^+^ and [Cu^II^(NH_3_)_3_(X)]^+^ at equilibrium are 65% and 35%, respectively. Considering that we have 16% Cu^I^ and 84% Cu^II^ before exposure to NH_3_ (see section 3.1), this means that approximately 58% of the amount of the copper ions in the μ-η^2^,η^2^-peroxo diamino dicopper(II) complexes are reduced to Cu^I^.

#### 3.2.2 Reactivity toward NH_3_: Interpretation

The results described above indicate that NH_3_ is reacting with the μ-η^2^,η^2^-peroxo diamino dicopper(II) complexes, breaking the copper dimer and reducing a consistent fraction of the Cu^II^ ions to Cu^I^. No N_2_ is observed during this reaction, in agreement with the fact that direct oxidation of ammonia (eq 2), only occurs on Cu-CHA at higher temperature.^50^
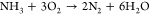
2The XANES and UV-Vis spectra (light blue curves in Figure 6a,d) are consistent with the presence of Cu^II^ ions in a pseudo-square-planar geometry, similar to the [Cu^II^(NH_3_)_3_(OH)] ^+^ species predicted by Paolucci et al. and experimentally observed by Borfecchia et al.^7,19^ We could thus hypothesize that the corresponding Cu^II^ ions are in the form of a superoxo amino [Cu^II^(NH_3_)_3_(OO*)]^+^ complex, as depicted in Figure 6e. This geometry is consistent with the relatively high intensity of the low-*k* sub-lobe in EXAFS WT data (Figure 4f and curve 4′ in Figure 5), related to multiple scattering contributions from N/O ligand atoms.

The superoxo [Cu^II^(NH_3_)_3_(OO*)]^+^ complex could be formed by a one-electron transfer from the bridged peroxo group in the μ-η^2^,η^2^-peroxo diamino dicopper(II) complexes to one of the Cu^II^ ions, with consequent formation of Cu^I^ ions and of the superoxo ligand. The resulting Cu^I^ ions are thus stabilized as [Cu^I^(NH_3_)_2_]^+^. This could be rationalized with eq 3, which should result in the reduction of 50% of the Cu^II^ ions in the dimer to Cu^I^.
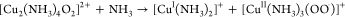
3Our XANES linear combination fitting indicates a higher efficiency of the Cu^II^-to-Cu^I^ reduction with NH_3_ (ca. 58% of Cu^II^ reduced) with respect to what is expected on the basis of eq 3. This could be related to a further reduction of the superoxo [Cu^II^(NH_3_)_3_(OO*)]^+^ complexes by the available NH_3_ present in the system, with formation of [Cu^I^(NH_3_)_2_]^+^. Interestingly, DFT simulations using the M06-HF-D3 functional predict that reaction 3 is exothermic at the experimental conditions. The computed variation of internal energy is −4.6 kJ/mol; see SI for further details. Despite the clear indications provided by the linear combination fitting and DFT simulations, the associated uncertainties are too high to use them as an ultimate proof for the reaction 3, so this may be investigated in further works. Interestingly, the reaction tentatively proposed in (3) could provide clues as to the origin of the observed NH_3_-inhibition effect and negative apparent NH_3_ rate orders, observed by different authors.^12,51,52^

#### 3.2.3 Reactivity toward NO: Experimental Evidence

The reaction of the μ-η^2^,η^2^-peroxo diamino dicopper(II) complexes with NO results in the separation of the copper centers, as shown by EXAFS-WT analysis (see above, Figure 4e). This is accompanied by some formation of N_2_, as monitored by online mass spectrometry (see SI, Figure S18). Due to the used experimental setup, the acquired mass spectrometry data are not accurate enough to be used for quantitative considerations. This section provides information about the dynamics of the reaction and summarizes the main experimental findings.

**Figure 7:**
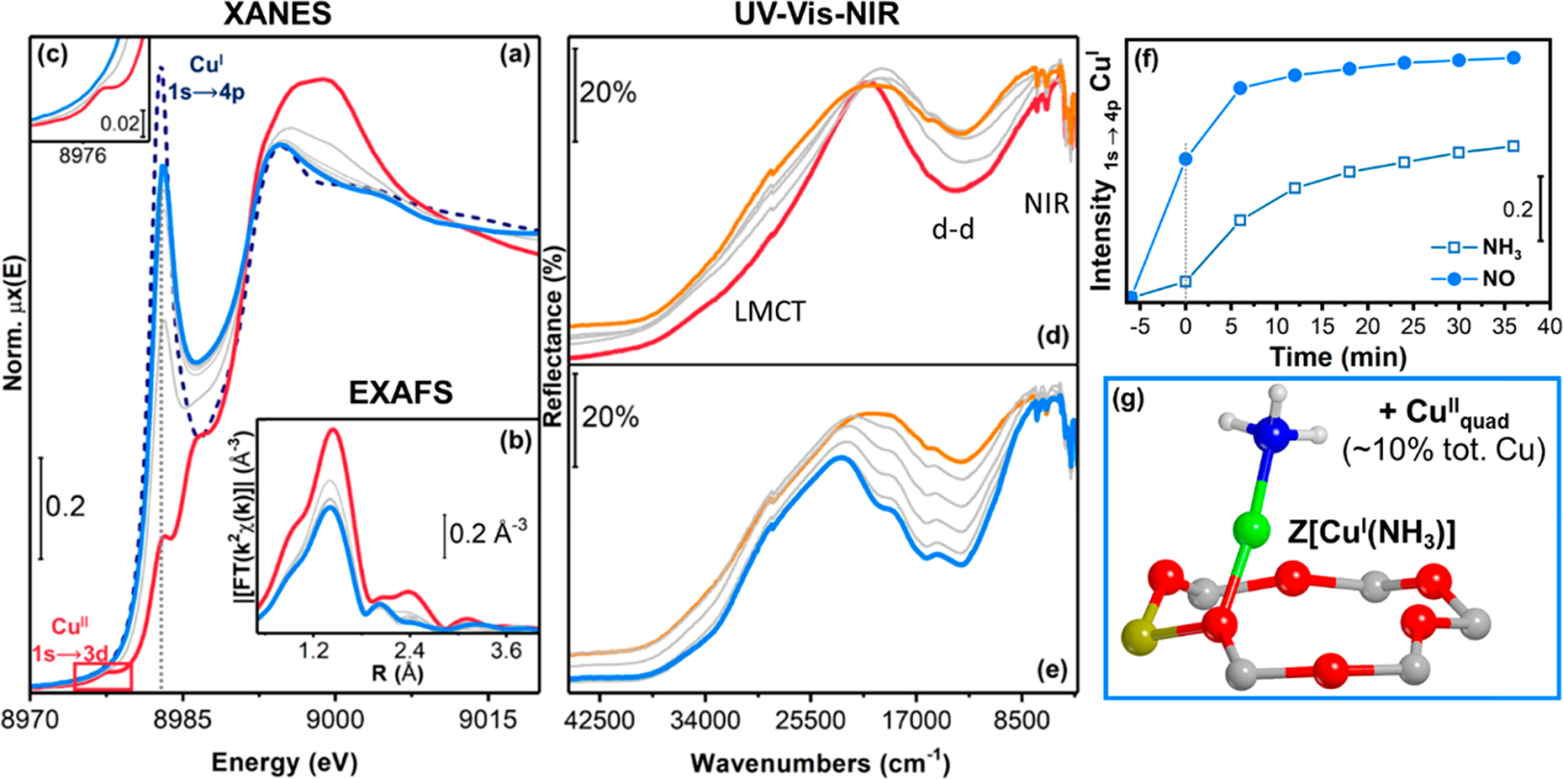
Exposure to NO/He at 200 °C of Cu-CHA catalyst after step 3. (a) Cu K-edge XANES spectra. (b) Phase-uncorrected *k*^2^-weighted FT EXAFS curves. (c) Magnification of Cu^II^ 1s→3d transition. (d) Initial and (e) subsequent evolution of DR UV-Vis-NIR spectra. Red thick line: spectrum collected in step 3 (same as Figures 2 and 3); light blue thick line: after of NO/He exposure; gray thin lines: intermediates; dashed dark blue line: step 2 (NO/NH_3_ at 200 °C after pretreatment in O_2_, same as dark blue in Figures 2 and 3); orange thick line is the final spectrum in the panel (d) and initial one in the panel (e). (f) Temporal evolution of the intensity of the Cu^I^ 1s →4p transition at ∼8982.5 eV during exposure to NO or NH_3_ after step 3. (g) Illustration of Cu species proposed to be formed upon reaction with NO. Atom color code: Cu, green; H, white; O, red; N, blue; Si, yellow; Al, pink.

Figure 7 reports the XAS and UV-Vis-NIR spectra collected when contacting the μ-η^2^,η^2^-peroxo diamino dicopper(II) complexes formed in step 3 with NO at 200 °C. The changes in the pre-edge features at the Cu K-edge in XANES reveals a fast and effective Cu^II^-to-Cu^I^ transformation. The characteristic 1s→4p transition at ∼8982.5 eV reappears in the spectrum (Figure 7a), and the weak 1s→3d transition at 8977.3 eV, indicative of a Cu^II^ species, disappears (Figure 7c). To illustrate the differences in the formation of Cu^I^ species in the reactions of the μ-η^2^,η^2^-peroxo diamino dicopper(II) complexes with NO and NH_3_, we have compared the temporal evolution of the 1s→4p transition at ∼8982.5 eV in the two cases (Figure 7f). These data show that the reduction of the μ-η^2^,η^2^-peroxo diamino dicopper(II) complex is faster and more efficient with NO than with NH_3_, since the Cu^I^ peak reaches about 76% of the intensity observed for the fully reduced reference state, i.e., [Cu^I^(NH_3_)_2_]^+^, as formed in step 2 (dark blue dashed line in Figure 7a) after only 5 min, stabilizing at about 84% after 35 min. In the case of reduction in NH_3_, the intensity is about 43% after 5 min, and reaches about 62% after 35 min. We note that the intensity of the Cu^I^ rising-edge peak can be affected by the geometry of Cu complexes.^43^ However, even though these data cannot be used to obtain precise kinetics of the reaction, they clearly show a difference in the reduction behavior in the two cases.

In the DR UV-Vis-NIR spectra, the reaction with NO is visible in the symmetrical d-d absorption at around 13850 cm^–1^, corresponding to the Cu^II^ sites in a pseudo-square-planar geometry in the μ-η^2^,η^2^-peroxo diamino dicopper(II) complex. In the first minutes of NO exposure, the intensity reaches a minimum (Figure 7d, from red to orange curve), followed by the development of a complex absorption with maxima at 20 000, 16 350, 13 300, and 10 600 cm^–1^ (shoulder), associated with Cu^II^ in a different local environment (Figure 7e, from orange to light blue). These new features resemble the typical “quadruplet”, as often observed in Cu-CHA samples after pretreatment in O_2_ (see Figure 2 and SI, Figure S14), which are assigned to the formation of a variety of monomeric and multimeric framework-coordinated Cu^II^ ions, such as Z[Cu^II^(OH)]/Z[Cu^II^(OO*)] sites etc.^26,33,34^ This indicates the formation of some oxidic Cu^II^ species during the reaction. The LMCT absorption between 27 000 and 31 000 cm ^–1^ (Figure 7d), related to the bridged peroxo groups in the μ-η^2^,η^2^-peroxo diamino dicopper(II) complex, also shows a rapid decrease in intensity in the reaction with NO. The subsequent change in the geometry of remaining Cu^II^ sites is reflected in a small blue-shift in the LMCT position (Figure 7e). We note that the XANES data do not reveal the presence of a Cu^II^ species after reaction with NO, suggesting that the observed Cu^II^ fraction remains below the XAS detection limit under our experimental conditions, which is estimated at around 10% of the total Cu content. This would then indicate that DR UV-Vis is very sensitive to the formation of this oxidic Cu^II^ species, due to the strong influence of the local geometry on the corresponding extinction coefficient.

The structure of the Cu^I^ ions formed during the reaction of μ-η^2^,η^2^-peroxo diamino dicopper(II) complex with NO is different from those observed after reduction in NO/NH_3_ at 200 °C or in the reaction with NH_3_. The shape of the XANES rising-edge and white-line peaks clearly differ from those of the linear [Cu(NH_3_)_2_]^+^ complexes, as indicated by the light blue and dotted dark blue curves in Figure 7a, and those of ligand-free, framework-coordinated ZCu^I^.^25^ Overall, the Cu K-edge XANES resembles that observed after desorption of NH_3_ (see SI, Figure S15), which has been assigned to framework-coordinated linear Cu^I^ amino complexes, Z[Cu^I^(NH_3_)].^19^ This assignment is supported by the NH_3_/NH_4_^+^ vibrational modes still present in the NIR region (Figure 7d and e) and by the decrease observed in the first-shell peak in EXAFS (Figure 7b), indicating a change from a four- to a two-fold coordination of Cu (Figures 4e and 7g). The broadening of the second and third shell regions can be moreover connected to the relatively high degree of freedom (in terms of bond length and angles) of the proposed Z[Cu^I^(NH_3_)] entities with respect to [Cu(NH_3_)_2_]^+^ or “bare” ZCu^I^ species.^6,10,19^

#### 3.2.4 Reactivity toward NO: Interpretation

The results in Figure 7d show that NO reaction causes a fast disaggregation of the side-on μ-η^2^,η^2^-peroxo diamino dicopper(II) complexes. The Cu^II^ species are almost completely reduced to Cu^I^, while the bridging peroxo groups are consumed and N_2_ is formed. The formation of N_2_ is fast (see SI, Figure S18), indicating that μ-η^2^,η^2^-peroxo diamino dicopper(II) complexes are very reactive toward NO. These results provide experimental support for the conclusion from DFT calculations, that NO facilitates the dissociation of the O–O bond in oxygen.^4,13,15,16^

According to our interpretation of the XANES results (Figure 7a), the Cu^I^ species after reaction with NO consists of framework-coordinated Z[Cu^I^(NH_3_)] moieties, implying that each Cu ion in the dimer loses only one amino ligand during the reduction. Starting from the μ-η^2^,η^2^-peroxo diamino dicopper(II) complexes, the NH_3_-SCR reaction requires stoichiometrically two NH_3_ molecules per Cu for a complete reduction of the Cu^II^,^8^ and therefore the second NH_3_ molecule is a nonligand NH_3_. In a recent DFT study, it is proposed that the NH_3_-SCR reaction proceeds via the decomposition of HONO and H_2_NNO intermediates to N_2_ and H_2_O, over Brønsted NH_4_^+^(or H^+^/NH_3_(g)) sites.^13^ This would be a way to include a second nonligand NH_3_ molecule in the SCR cycle, but such a role of Brønsted sites in the NH_3_-SCR cycle still needs experimental verification.^53^

Even though the XANES results point to a complete reduction of the Cu^II^, a minor fraction of Cu^II^ is still present after reaction with NO, which remains below the detection limit of XANES under our experimental conditions. The presence of these Cu^II^ moieties in the XAS experiment is indicated by the small amount of N_2_ that is formed upon adding NH_3_ to the NO feed after step 4, to restore the fully reduced state consisting of [Cu(NH_3_)_2_]^+^ complexes (SI, Figure S19). The formation of N_2_ indicates that some reduction of Cu^II^ takes place, thus proving that the reduction with NO alone was not complete. We expect the amount of this residual Cu^II^ fraction to depend on the Cu content and Si/Al ratio of the Cu-CHA material.

The features in the UV-Vis observed at 20 000, 16 350, 13 300, and 10 600 (sh) cm^–1^ after the reaction with NO is completed resemble the UV–Vis “quadruplet”, that is often observed for Cu^II^ in Cu-CHA and other small pore zeolites.^26,33−36,54^ These features have been assigned to Cu^II^ species attached to the zeolite framework, such as Z[Cu^II^(OH)], Z[Cu^II^(OO*)], framework-coordinated Cu dimers with O/OH bridging moieties, or even larger Cu clusters.^34^ This suggests that the Cu^II^ species that remains after reaction of the μ-η^2^,η^2^-peroxo diamino dicopper(II) complex with NO is attached to the zeolite framework as well. We also note that the high intensity of these features is comparable to that observed in fully oxidized Cu-CHA samples (see SI, Figure S14), despite the low fraction of Cu^II^. This puzzling finding could be related to the fact that this spectroscopic feature is the result of a variety of Cu^II^ ions with similar but not identical local environments affecting the d-splitting,^55,56^ as recently predicted by Li et al.^34^

## 4 Conclusions

We have studied the activation of oxygen over the mobile linear [Cu(NH_3_)_2_]^+^ complexes in a Cu-CHA catalyst for NH_3_-SCR (Si/Al ratio = 15 and 2.6 wt% Cu), which is a crucial step in the NH_3_-SCR reaction, by combining X-ray absorption spectroscopy, and diffuse reflectance UV-Vis-NIR spectroscopy.

The reaction of the linear [Cu(NH_3_)_2_]^+^ complexes with O_2_ at 200 °C results in the formation of a side-on μ-η^2^, η^2^-peroxo diamino dicopper(II) complex ([Cu_2_(NH_3_)_4_O_2_]^2+^) as shown in Figure 1b, indicating a reaction of O_2_ with a pair of [Cu(NH_3_)_2_]^+^ complexes. About 84% of the Cu present is oxidized by O_2_, the remaining 16% stays present as linear [Cu(NH_3_)_2_]^+^ species. The structure of the [Cu_2_(NH_3_)_4_O_2_]^2+^ complex also indicates that an O–O bond is retained in this reaction. We have also successfully applied a wavelet transform analysis of the EXAFS data to identify Cu–Cu scattering contributions after the reaction with O_2_, providing unprecedented direct spectroscopic evidence for the formation of Cu-pairs in the NH_3_-SCR reaction.

The [Cu_2_(NH_3_)_4_O_2_]^2+^ complexes show a different reactivity toward NH_3_, NO, or a mixture of NO and NH_3_ at 200 °C. The reaction with a mixture of NO and NH_3_ leads to a complete reduction of the [Cu_2_(NH_3_)_4_O_2_]^2+^ complexes with evolution of N_2_, and the linear [Cu(NH_3_)_2_]^+^ complexes are restored, confirming that the [Cu_2_(NH_3_)_4_O_2_]^2+^ complex plays a role in the NH_3_-SCR reaction cycle.

In the reaction of the [Cu_2_(NH_3_)_4_O_2_]^2+^ complex with NH_3_, in the absence of NO, about 58% of the Cu^II^ species is reduced to Cu^I^, while no N_2_ is formed. The [Cu_2_(NH_3_)_4_O_2_]^2+^ complexes dissociate and a variety of mononuclear Cu complexes is formed, consisting of Cu^I^ and Cu^II^ species with NH_3_ and oxidic ligands.

In the reaction with NO, some N_2_ is formed, and the [Cu_2_(NH_3_)_4_O_2_]^2+^ complexes are almost completely reduced. The formation of N_2_ indicates that the NH_3_-SCR cycle involves a reaction of the [Cu_2_(NH_3_)_4_O_2_]^2+^ complex with NO. The Cu^I^ species formed in this reaction is probably a Z[Cu^I^(NH_3_)], which is attached to the zeolite framework. A minor part (<10%) of the Cu remains in a Cu^II^ state, due to a lack of reactive NH_3_ in the catalyst. Addition of NH_3_ at this stage leads to the restoration of the linear [Cu(NH_3_)_2_]^+^ complexes with N_2_ evolution, further confirming the role of the reaction of NO with the [Cu_2_(NH_3_)_4_O_2_]^2+^ complexes in the NH_3_-SCR reaction cycle.

## Supporting Information Available

Experimental details on spectroscopic experiments; computational details; EXAFS fitting methods and results; wavelet transform (WT) analysis of EXAFS spectra, methods and additional results; additional in situ spectroscopy results; mass spectrometry results (PDF)
