# Supplementary material for: Structure and Reactivity of Oxygen-Bridged Diamino Dicopper(II) Complexes in Cu-Ion-Exchanged Chabazite Catalyst for NH3-Mediated Selective Catalytic Reduction
Source: J Am Chem Soc. 2020 Aug 24;142(37):15884–96. doi: 10.1021/jacs.0c06270 (PMC8011910; doi:10.1021/jacs.0c06270)
Supplement: Supplementary file 1 — ja0c06270_si_001.pdf [file ja0c06270_si_001.pdf]

Supporting Information

Structure and Reactivity of Oxygen-Bridged Diamino Dicopper(II) Complexes in Cu-Ion-Exchanged Chabazite Catalyst for NH_3-Mediated Selective Catalytic Reduction

Chiara Negri, [a †§] Tommaso Selleri, [b†‡] Elisa Borfecchia, [a] Andrea Martini, [a,c] Kirill A. Lomachenko, [d] Ton V.W. Janssens, [e] Michele Cutini, [a] Silvia Bordiga,[a] and Gloria Berlier [a*]

[a] *Department of Chemistry and NIS Centre, University of Turin, Via Giuria 7, Turin, 10125 (I)*

[b] *Dipartimento di Energia, Laboratorio di Catalisi e Processi Catalitici, Politecnico di Milano, Via La Masa 34, I-20156, Milano, (I)*

[c] *Smart Materials, Research Institute, Southern Federal University, Sladkova Street 174/28, 344090 Rostov-on-Don, Russia.*

[d] *European Synchrotron Radiation Facility, 71 Avenue des Martyrs, CS 40220, 38043 Grenoble Cedex 9, France*

[e] *Umicore Denmark ApS, Nøjsomhedsvej 20, Kgs. Lyngby, 2800 (Denmark)*

[†] These first authors contributed equally to this article.

[§] Present address: *Department of Chemistry, Center for materials science and nanotechnology, University of Oslo, Sem Sælands vei 26, 0371 Oslo, Norway*

[‡] Present address: *European Commission Joint Research Centre, 21027 Ispra, Italy*

Contents

1 Methods 3

1.1 In situ spectroscopies 3

1.2 Experiment description 3

1.3 Computational details 3

2 EXAFS fitting methods and results 8

2.1 EXAFS analysis for step #1 (pre-treatment in O_2) 8

2.2 EXAFS analysis for step #2 (reduction in NH_3/NO) 10

2.3 Test EXAFS fit for step #3 (oxidized in O_2) considering the same fw-Cu^{II} model employed for step #1 11

2.4 EXAFS analysis for oxidized state (step #3) considering $[Cu_2(NH_3)_4(O_2)]^{2+}$ models 12

2.4.1 Details on fitting strategy and shell model adopted for EXAFS analysis 13

2.4.2 Results from single-component EXAFS fits based on DFT-optimized candidate geometries. 13

2.4.3 Multi-component fit considering peroxo side-on $[Cu_2(NH_3)_4(O_2)]^{2+}$ and $[Cu^I NH_3)_2]^+$ complexes 15

3 Wavelet Transform (WT) analysis of EXAFS spectra: methods and additional results 17

3.1 Relevance and methods for EXAFS WT analysis 17

3.2 Comparative overview of in situ EXAFS spectra by conventional visualization in k-space and R-space 18

3.3 Full-range Wavelet Transform representation of in situ EXAFS spectra 18

4 Additional *in situ* spectroscopy results 19

5 Mass spectrometry results obtained during *in situ* XAS experiments 23

1 Methods

1.1 *In situ spectroscopies*

X-ray Absorption Spectroscopy (XAS) data were collected at the BM23 beamline[1] of the European Synchrotron Radiation Facility (ESRF, Grenoble, France) using the Microtomo reactor cell.[2] The Cu-CHA catalyst was prepared in the form of a self-supporting pellet (~ 100 mg/1.3 cm^2, resulting in an edge jump $\Delta\mu x$ = 0.6 for a total absorption after the edge of μx = 2.5). Cu K-edge XAS measurements were performed in transmission mode, employing a double-crystal Si(111) monochromator for the incident energy scan, a pair of flat Si mirrors at 2.5 mrad angle for harmonics rejection, and ionization chambers to detect incident (I_0) and transmitted ($I_{1,2}$) photons. A Cu metal foil was measured simultaneously using a third ionization chamber I_2, for energy calibration purposes.[3] XAS spectra of ~ 20 min each (energy range 8800 – 9965 eV; pre-edge region energy step = 5 eV, edge region energy step = 0.3 eV, constant k-space sampling Δk = 0.035 Å^{-1} in the EXAFS region; acquisition time of 1 s/point in the whole energy range) were measured at stationary conditions. The three corresponding $\mu(E)$ curves were averaged after checking reproducibility among the consecutive acquisitions.

The gas composition of the outlet gas from the reactor has been continuously monitored by means of a mass spectrometer.

The Athena software (Demeter package)[4] was used to align XAS data by using the corresponding Cu metal foil spectra and for normalization to unity of the edge jump. The same program was used for the extraction of the $\chi(k)$ function. R-space FT-EXAFS spectra were obtained by calculating the Fourier transform of the $k^2\chi(k)$ functions in the (2.4 – 12.4) Å^{-1} k-range.

Detailed descriptions of the methods adopted for DFT-assisted EXAFS fitting as well as for EXAFS WT analysis can be found in the following Sections 2 and 3, respectively.

DR UV-Vis-NIR spectra were recorded in the 2500-200 nm range (50000 – 4000 cm^{-1}) at 1 nm resolution on a Varian Cary 5000 spectrophotometer, equipped with a R928 PMT UV-Vis detector and a cooled PbS photocell NIR detector. Spectra were collected with a Praying Mantis® element, coupled with a low temperature (LT) reaction chamber. Measurements were carried out on sieved samples (150 ÷ 300 μm), pelletized with a hydraulic press and successively crushed and sieved. Teflon powder in the same LT cell was measured as a reference. Spectra are reported as relative reflectance (R%), defined as:

$$R_{\%} = R_{sample}/R_{reference} \cdot 100$$

1.2 *Experiment description*

The gas flow used in XAS and DR UV-Vis-NIR experiments was 100 and 50 ml/min, respectively. In all the experiments, the measured catalyst was first treated in O_2 at 400 °C for 60 min (heating rate 5 °C/min), then cooled to 200 °C (cooling rate 3°C/min) in O_2 and treated with 1000 ppm NO and 1000 ppm NH_3 in He diluent. After reaching steady state conditions (typically 90 min for both UV-Vis and XAS) the system purged with N_2 was then exposed to 10% O_2 in He. After reaching steady state conditions (typically 30 min) 1000 ppm NH_3 or 1000 ppm NO (rest He) were dosed in separate experiments. The reversibility of the cycle was then checked by adding NO or NH_3 (1000 ppm, rest He) to the feed after reaching steady conditions. The measurement cells inlet lines were heated to 130 °C to minimize NH_3 adsorption in the equipment. All reported data were measured at 200 °C.

1.3 *Computational details*

Investigating with molecular simulations the $[Cu_2(NH_3)_4O_2]^{2+}$ complex within a zeolite framework is a challenging task. The isolated complex has been already investigated in refs. [5-6], using several methodologies spanning from DFT to post-Hartree-Fock (HF) with substantial disagreement between the different levels for most cases. Recently, the potential energy path of direct O_2 dissociation on $[Cu(NH_3)_2]^+$ pairs in CHA has been investigated with DFT simulations,[7] also assessing the critical role of the methodogy.[8]

In this work we employed DFT simulations with the purpose of providing reasonable structures of the different conformers of the $[Cu_2(NH_3)_4O_2]^{2+}$ complex for comparing with experimental data and EXAFS analysis. Considered the complexity of the system under study we have followed the idea of adding and error bar to the DFT simulations by varying the amount of exact HF exchange energy in the functional so tuning the electron delocalization, as presented in Ref. [9] The extreme behavior of over-delocalization is given by the local density approximation, which describes the homogeneous electron gas, delocalizing electrons like in a metal. On the other extreme, HF is built to promote electron-pairing, leading to an over-localization of electrons.

We run DFT simulation using M06 suit of functionals which allows having several similar functionals with different HF exchange energy contributions. We employed the M06-L functional,[10] the M06-HF functional,[11] and the M06 functional.[12] The M06-L/M06/M06-HF functional have 0%/27%/100% of HF exchange energy. In line of principle the M06-L and M06-HF approaches represent limit methodologies for the electron density delocalization within the class of M06 functionals.

All calculations were performed within the UDFT approach, using the Gaussian 09 program.[13] Simulations are corrected for dispersion by the D3 scheme.[14] We employed the Def2-TZVP basis set as coded in the Gaussian 09 suit.[15] Basis set convergence with a larger Def2-QZVP basis has been assessed. The integral grid size is set to ultrafine and the geometry optimization convergence to tight. Frequency estimations are carried out after geometry optimization to check if the geometry-optimized structure is a true minimum of the potential energy surface. Solvation effects were taken into account using the PCM polarized continuum model, to mimic the ammonia-rich environment within the zeolite framework.[16] Ammonia solvent has been modelled using the default PCM values then varying the dielectric constant of the solvent to that of liquid ammonia to 14.01.[17] All complexes have charge +2 and are spin polarized with ground state with triplet, singlet bi-radical or close shell singlet character.

We have investigated several conformation/wave-function-character combinations, using M06-HF-D3 and M06-L-D3 functionals. The relaxed geometries images are reported in Scheme **S1** and S2; geometry and energy details in Table **S1**, Scheme **S3**, **S4** and Figure **S1**. Regarding the conformations for the $[Cu_2(NH_3)_4O_2]^{2+}$ complex (different columns in Scheme **S1-S4**), we have considered: i) the cis and trans end-on peroxo; ii) the side-on peroxo with Cu and O atoms on the same plane (IP) and out of plane (OP); iii) the bis-μ-oxo with Cu and O atoms in plane (IP) and out of plane (OP). The wave-function (different rows in Scheme **S1-S4**) can be characterized by: i) a triplet spin state (OST); ii) a singlet spin state with paired electrons on each Cu atoms (OSS); iii) a close shell singlet spin state (CSS). Blank cells in Schemes **S1-S2** indicate the not stable conformation/wave-function-character combinations.

From the data analysis, the following considerations emerge:

1) The energy ranking between conformers is method dependent, see Figure **S1**. The use of a hybrid functional stabilizes the bi-radical wave function which is typical of the peroxide species. Therefore, using the M06-HF/M06-L functional the oxide is higher/lower in energy of 395.7/17.4 $kJ \cdot mol^{-1}$ than the side-on peroxide. The relative stability among the two structures changes as a function of the type of functional employed. For instance, using the M06 functional, the oxide specie is computed to be only +41.5 $kJ \cdot mol^{-1}$ more unstable than the side-on peroxide. Therefore, excluding the HF exact exchange from the functional the most stable copper complex conformation is the oxide, see Chen et al.[8]
2) On the geometry side, we have considered the results of the M06-L and M06-HF as limits situations for the geometrical parameters for the complex. We have considered the most stable conformation geometries for each column of Scheme **S3** and **S4**, for the M06-L and M06-HF methods (green rectangles). The limit geometries for the different conformations of the complex are reported in Table **S1**. Interestingly, we computed the relaxed geometry for most of the copper complexes using the M06 functional. The interatomic distances computed at this level fall within the limits reported in Table 1, confirming the validity of this approach in adding an error bar to DFT results.

The experimental EXAFS fitting analysis was run starting from the following DFT (M06-HF) relaxed structures: side-on peroxo complex (OP) with OSS wave-function, *trans* end-on peroxo with OST wave-function, and bis-μ-oxo IP with CSS wave-function.

Regarding the equation (3) reported in the main text, we have computed the thermodynamics of the reaction using the M06-HF-D3 level of theory. We considered the stable wave function for reactants and products. In details, the (side-on)-peroxo dicopper complex is computed as singlet bi-radical, ammonia as closed shell singlet, $[Cu^{I}(NH_3)_2]^+$ as closed shell singlet, and $[Cu^{II}(NH_3)_3(OO^{\bullet})]^+$ as triplet.

The internal energy of each reactants and products is computed directly by the Gaussian 16 program and it printed out in a vibrational frequencies calculation as sum of electronic and thermal energies.

**Table S*1*.** Geometrical parameters computed at the M06-L and M06-HF levels	Bond length (Å)		
	Cu-Cu	Cu-O	O-O
trans (end-on) μ-1,2-peroxo dicopper (II)	4.149 ÷ 4.300	1.860 ÷ 2.113	1.270 ÷ 1.449
(side-on) μ-η^2,η^2-peroxo dicopper (II)	3.059 ÷ 3.597	1.910 ÷ 1.983	1.416 ÷ 1.508
bis-μ-oxo dicopper (III)	2.644 ÷ 2.872	1.772 ÷ 1.870	2.265 ÷ 2.359

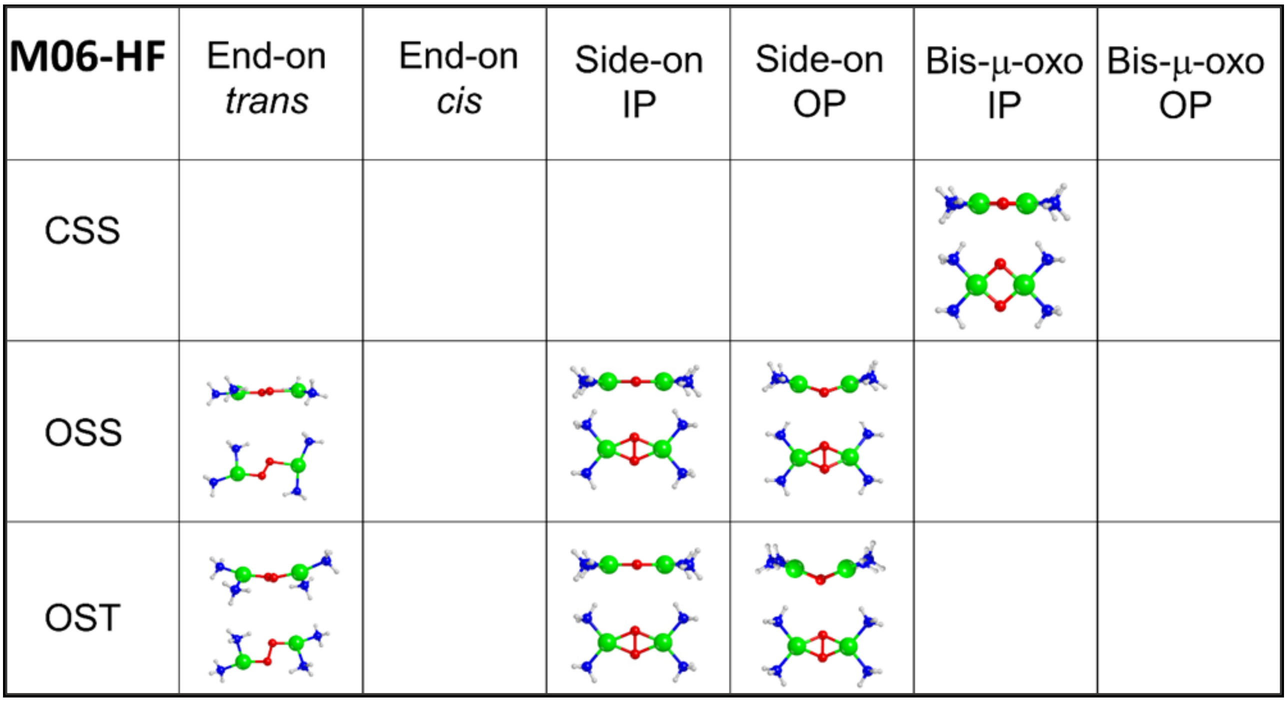

Scheme S1. *Images (side and top views) of the M06-HF-D3 relaxed* $[Cu_2(NH_3)_4O_2]^{2+}$ *complex for different conformation/wave-function-character combination. Atom colour code: Cu, green; H, white; O, red; N, blue.*

**M06-L**	End-on *trans*	End-on *cis*	Side-on IP	Side-on OP	Bis-μ-oxo IP	Bis-μ-oxo OP
CSS			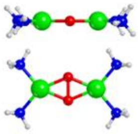		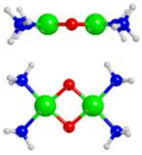	
OSS	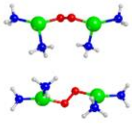		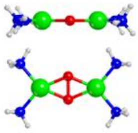			
OST	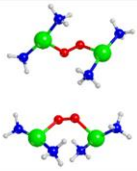			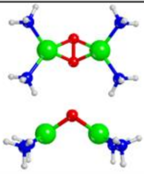		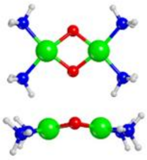

Scheme S2. *Images (side and top views) of the M06-L-D3 relaxed [$Cu_2(NH_3)_4O_2]^{2+}$ complex for different conformation/wave-function-character combination. Atom colour code: Cu, green; H, white; O, red; N, blue.*

**M06-HF**	End-on *trans*	End-on *cis*	Side-on IP	Side-on OP	Bis-μ-oxo IP	Bis-μ-oxo OP
CSS					Charge Cu=1.68<br>Cu-Cu d.= 2.644<br>Cu-O d.= 1.772<br>O-O d.=2.359<br>Spin Cu=0.0/0.0<br>ΔE=395.7	
OSS	Charge Cu=1.56/1.56<br>Cu-Cu d.= 4.300<br>Cu-O d.= 1.860<br>O-O d.=1.449<br>Spin Cu=0.90/-0.90<br>ΔE=81.20		Charge Cu=1.57<br>Cu-Cu d.= 3.597<br>Cu-O d.= 1.950<br>O-O d.=1.508<br>Spin Cu=0.82/-0.82<br>ΔE=1.8	Charge Cu=1.54<br>Cu-Cu d.= 3.400<br>Cu-O d.= 1.955<br>O-O d.=1.504<br>Spin Cu=0.81/-0.81<br>ΔE=0.0		
OST	Charge Cu=1.56/1.56<br>Cu-Cu d.= 4.295<br>Cu-O d.= 1.860<br>O-O d.= 1.449<br>Spin Cu=0.90/0.90<br>ΔE=81.24		Charge Cu=1.59<br>Cu-Cu d.= 3.613<br>Cu-O d.= 1.958<br>O-O d.=1.512<br>Spin Cu=0.84/0.84<br>ΔE=10.7	Charge Cu=1.54<br>Cu-Cu d.= 3.330<br>Cu-O d.= 1.961-1957<br>O-O d.=1.504<br>Spin Cu=0.82/0.82<br>ΔE=4.0		

Scheme S3. *Geometrical and electronic features of the M06-HF-D3 relaxed [$Cu_2(NH_3)_4O_2]^{2+}$ complex for different conformation/wave-function-character combination.*

**M06-L**	End-on *trans*	End-on *cis*	Side-on IP	Side-on OP	Bis-μ-oxo IP	Bis-μ-oxo OP
CSS			Charge Cu=0.77<br>Cu-Cu d.= 3.532<br>Cu-O d.= 1.910<br>O-O d.= 1.452<br>Spin Cu=0.0/0.0<br>ΔE=17.4		Charge Cu=1.17<br>Cu-Cu d.= 2.746<br>Cu-O d.= 1.799<br>O-O d.= 2.326<br>Spin Cu=0.0/0.0<br>ΔE=0.0	
OSS	Charge Cu=0.75<br>Cu-Cu d.= 4.379<br>Cu-O d.= 1.862<br>O-O d.=1.326<br>Spin Cu=0.37/-0.37<br>ΔE=83.5		Charge Cu=0.45<br>Cu-Cu d.= 3.534<br>Cu-O d.= 1.910<br>O-O d.= 1.453<br>Spin Cu=0.1/-0.1<br>ΔE=17.5			
OST	Charge Cu=0.60<br>Cu-Cu d.=4.149<br>Cu-O d.=2.097-2.113<br>O-O d.= 1.270<br>Spin Cu=0.25/0.25<br>ΔE=20.2			Charge Cu=+1/+1<br>Cu-Cu d.= 3.059<br>Cu-O d.= 1.983<br>O-O d.=1.416<br>Spin Cu=0.44/0.44<br>ΔE=22.5		Charge Cu=0.5/0.5<br>Cu-Cu d.= 2.872<br>Cu-O d.= 1.870-1.83<br>O-O d.= 2.265<br>Spin Cu=0.1/0.3<br>ΔE=62.6

Scheme S4. *Geometrical and electronic features of the M06-L-D3 relaxed $[Cu_2(NH_3)_4O_2]^{2+}$ complex for different conformation/wave-function-character combination.*

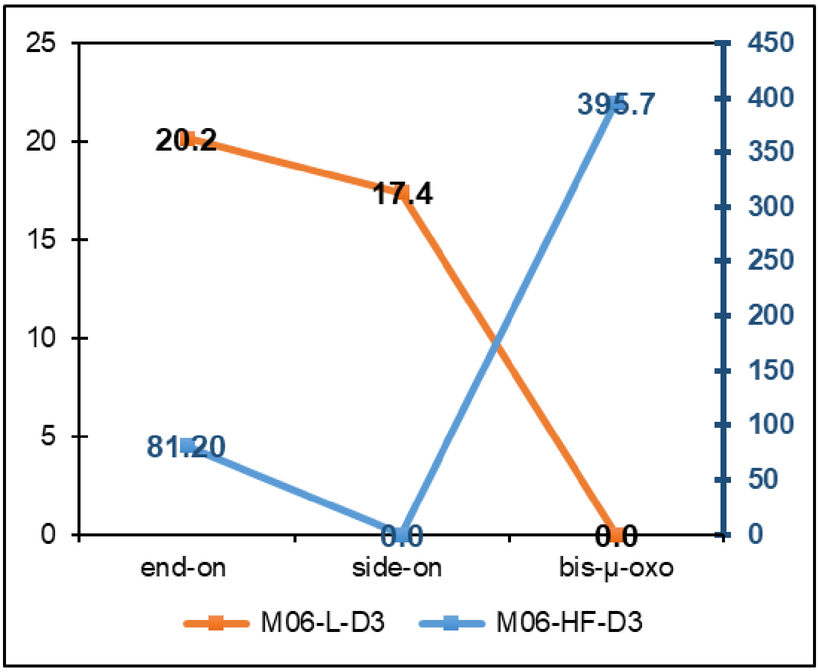

Figure S1. *Energy ranking for the different complexes at the M06-HF-D3 (right vertical axis) and M06-L-D3 (left vertical axis) levels. Energy in kJ·mol^{-1}.*

2 EXAFS fitting methods and results

2.1 EXAFS analysis for step #1 (pre-treatment in O_2)

A first step in our analysis consists in building up a suitable structural model to describe Cu(II) species initially present in the catalyst after pretreatment. Cu-speciation after high-temperature pretreatment in O_2 has been explored in several studies, both related to NH_3-SCR catalysis and direct methane to methanol conversion.[18-20] From these studies, it is evident that after pretreatment in O_2, a pool of framework-coordinated Cu(II) species exists in the catalyst, preferentially stabilized in the 8MR of the CHA zeolite.[21-22]

Together with previously reported evidences from Raman and UV-Vis spectroscopy,[19, 23] EXAFS WT results reported in this work directly shows the existence of Cu-Cu contributions already after step #1, pointing to the presence of multi-copper moieties involving a significant fraction of the Cu atoms present in the catalyst. Thus, multi-copper species are surely present after pretreatment, but the simultaneous presence of Cu(II) monomeric species cannot be ruled out.

All these species share some common, distinctive characteristics:

- The Cu(II) ion is coordinated to two oxygen atoms of the zeolite lattice (2 O_{fw}) while their first coordination sphere is completed by extra-framework O_{ef} ligands (1 O_{ef}, in the most common “tridentate” coordination motif).
- The Cu(II) ion is stabilized in proximity of a charge balancing zeolite Al atom, at ca. 2.75 Å from the metal center; all the other Si/O atoms of the framework fall at higher distances, typically > 3 Å. Such peculiar connectivity towards Al can be assumed as a fingerprint in the EXAFS of framework-coordinated Cu(II) species in CHA.

Conversely, DFT-optimized Cu-Cu separations in multicopper species undergoes important species-dependent variations, ranging e.g., from ca. 3.0 Å up to ca. 4.5 Å. Such indications fall in line with the broadness of the Cu-Cu sub-lobe along the R-direction in the WT map obtained after step #1. Notably, differences in Al siting will also result in perturbation of Cu-Cu distances, even considering the same chemical moiety.

In this rather heterogeneous scenario, it is not possible to select one, well-defined geometry as starting input for the EXAFS fitting of the spectrum obtained for the pre-treated catalyst. Hence, we have built up a suitable fitting model based on the structural characteristics conserved for most framework-coordinated Cu(II) species (fw-Cu^{II}) previously proposed in Cu-CHA, maintaining a certain degree of flexibility to account for fractional contribution from multi-copper species.

The adopted fitting model describes a prototypical tridentate fw-Cu(II) species, considering SS contributions calculated on the basis of a Z[Cu^{II}OH] site in the CHA 8MR. As illustrated in the first row of Table S2, first-shell contributions include two sub-shells of framework oxygen atoms, O_{ef} (N_{Oef} = 1) and O_{fw} (N_{Ofw} = 2), while the characteristic Cu-Al_{fw} contribution is included in the second coordination shell of Cu. These SS paths are parametrized with independent interatomic distances and Debye-Waller (DW) parameters for $O_{(ef+fw)}$ and Al_{fw} shells. At higher R, SS stemming from more distant Si and O atoms of the framework (fw) up to 4 Å (N_{fw} = 4) are included and parametrized considering a common contraction/expansion factor α_{fw} and DW factor σ^2_{fw} increasing as the square root of the distance $R_{eff,i}$ of the ith scattering atom from the absorber ($\Delta R_{fw,i} = \alpha_{fw} R_{eff,i}$, $\sigma^2_{fw,i} = ss_{fw} (R_{eff,i}/R_0)^{1/2}$, where R_0 denotes the shortest R_{eff} for the group of paths). Finally, a Cu-Cu_{ef} SS path with initial R_{eff} = 3.4 Å is included and refined with guessed coordination number N_{Cu} and interatomic distances R_{Cu}, while fixing the corresponding Debye-Waller factor σ^2_{Cu} to avoid excessively high correlations with the N_{Cu} fitting variable.

***Table S2**. Results of the fits executed on the k^2-weighted FT-EXAFS spectrum of Cu-CHA (transformed in the 2.4-13.0 $Å^{-1}$ k-range) pre-treated in O_2 at 400 °C and measured after cooling down to at 200 °C in O_2 (step #1, grey dashed curve in Figure 3, main text). The model of framework (fw)-coordinated Cu^{II} species used as initial guesses for the fitting procedure is also illustrated. Colour code in the structures: **white**: hydrogen; **red**: oxygen, **yellow**: aluminium, **grey**: silicon; **green:** copper. The atoms included in the*

fitting model are shown in ball-and-stick mode, while the others are visualized in stick mode. Parameter values kept fixed in the fit are underlined.

Exp.: Step #1 Pre-treatment in O_2
EXAFS fitting model: fw-Cu^{II}

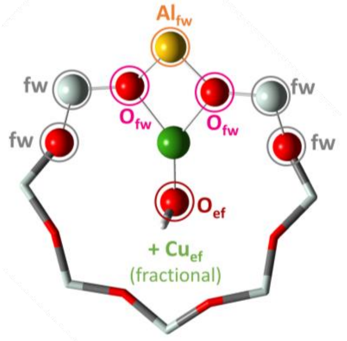

EXAFS parameters	*Best-fit values*
**R-space fitting range (Å)**	1.0-4.0
**$N°_{par}/N°_{ind}$**	10/19
**R_{factor}**	0.016
**S_0^2**	0.9
**ΔE (eV)**	-3 ± 1
**N_{O_ef} (Å)**	1
**N_{O_fw} (Å)**	2
**N_{Al_fw} (Å)**	1
**N_{Cu_ef} (Å)**	0.5 ± 0.3
**R_{O_ef} (Å)**	1.85 ± 0.04 (1.77)
**R_{O_fw} (Å)**	1.95 ± 0.01 (1.99)
**R_{Al_fw} (Å)**	2.71 ± 0.02 (2.75)
**R_{Cu_ef} (Å)**	3.36 ± 0.05 (3.40)
**α_{fw}**	- 0.01 ± 0.01
**σ^2_O (Å^2)**	0.004 ± 0.002
**$\sigma^2_{Al\_fw}$ (Å^2)**	0.009 ± 0.003
**$\sigma^2_{Cu\_ef}$ (Å^2)**	0.01
**ss_{fw} (Å^2)**	0.017 ± 0.008

The fit resulted in a good level of reproduction of experimental EXAFS spectrum for the pretreated catalyst at step #1 (see Figure S2) with physically meaningful values for all the refined parameters, as well as for their fitting errors (see Table S2). In general, beyond the first shell, rather high values of DW factors are obtained, which are however reasonable considering both the data collection at 200 °C and the high level of structural heterogeneity, as visually evidenced by WT analysis.

Considering the principal scattering contributions reported in Figure S2, we note how the second shell feature, peaking at ca. 2.3 Å in the phased-uncorrected FT spectra, receives substantial contribution from Cu-Al_{fw} path. At higher R-values, the spectral shape is determined by the superimposition of SS paths involving fw and Cu_{ef} atomic neighbors (refined with N_{Cu_ef} = 0.5 ± 0.3, thus indicating the involvement of ca. half of the Cu ions present into multi-copper configurations.

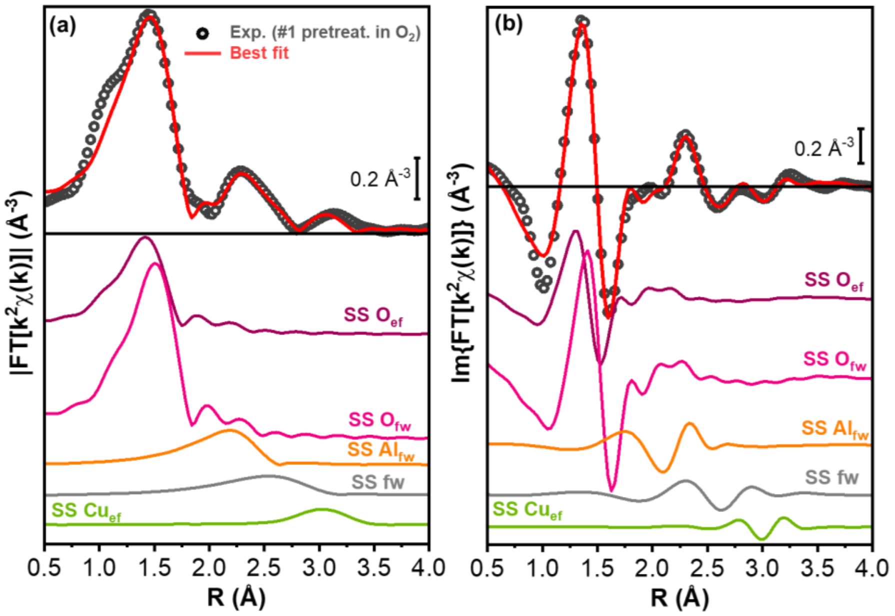

***Figure S2**. Phase uncorrected, modulus (a) and imaginary part (b) of the experimental and best fit FT EXAFS spectra for step #1 (pre-treated in O_2). The experimental data are shown as dark grey open circles and the best fits with red solid lines. The principal SS and contributions included in the fitting model are reported as solid lines vertically translated for sake of clarity.*

2.2 EXAFS analysis for step #2 (reduction in NH_3/NO)

To further corroborate the fact that after reduction in NO/NH_3 at 200 °C (step #2) virtually all the Cu present in the catalyst occurs as mobile $[Cu^I(NH_3)_2]^+$ complexes, we fitted the related EXAFS spectrum using DFT-optimized geometry of $[Cu^I(NH_3)_2]^+$ as starting input. The adopted fitting model includes SS paths from first-shell N atoms (N_N= 2, fixed) as well as intense collinear MS paths which involve the first-shell N atoms and provide a significant contribution in the 3.0-3.5 Å R-space range. All the SS and MS paths were refined using the same passive amplitude reduction factor (S_0^2) and the same energy shift parameter (ΔE). The fit successfully reproduced the experimental EXAFS spectrum for the reduced catalyst at step #2 (see Figure **S3**) with physically meaningful values for all the refined parameters, as well as for their fitting errors (see Table **S3**).

***Table S3.** Results of the fits executed on the k^2-weighted FT-EXAFS spectrum (transformed in the 2.4-13.0 Å^{-1} k-range) of Cu-CHA reduced in NO/NH_3/He at 200 °C (step #2, blue curve in Figure 3, main text). The model of $[Cu^I(NH_3)_2]^+$ complex used as initial guess for the fitting procedure is also illustrated. Colour code in the structural model: **white**: hydrogen; **blue**: nitrogen; **green:** copper. The atoms included in the fitting model are shown in ball-and-stick mode, while the others are visualized in stick mode.*

Exp. – Step #2 reduction in NO/NH_3
EXAFS fitting model – $[Cu^I(NH_3)_2]$+

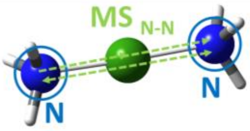

EXAFS parameters	*Best-fit values*
**R-space fitting range (Å)**	1.0-4.0
**$N°_{par}/N°_{ind}$**	4/19
**R_{factor}**	0.016
**S_0^2**	0.94 ± 0.05
**ΔE (eV)**	-2 ± 1
**N_N (Å)**	2
**<R_N> (Å)**	1.913 ± 0.004 (1.943)
**σ_N (Å^2)**	0.0041 ± 0.0006

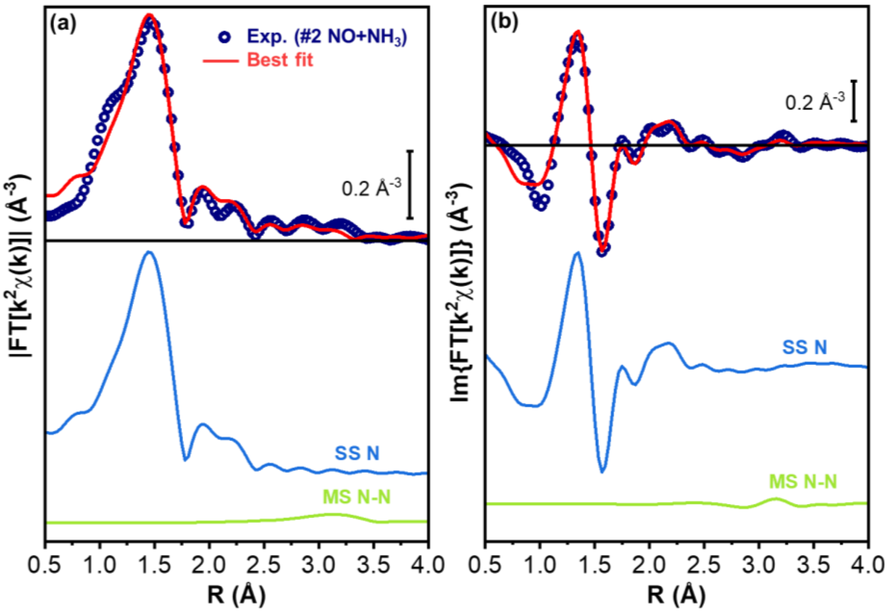

Figure S3*. Phase uncorrected, modulus (a) and imaginary part (b) of the experimental and best fit FT EXAFS spectra for step #3 (reduced in NO/ NH_3/He at 200 °C). The experimental data are shown as blue open circles and the best fits with red solid lines. The principal SS and MS contributions included in the fitting model are reported as solid lines vertically translated for sake of clarity.*

2.3 *Test EXAFS fit for step #3 (oxidized in O_2) considering the same fw-Cu^{II} model employed for step #1*

The results from EXAFS fits reported in previous sections 2.1 and 2.2 are herein employed to support the spectroscopy diversity and distinguishability of the catalyst state at step #1 and step #3, which are both characterized by dominant presence of Cu(II) species. To this aim, we have performed a test EXAFS fit using exactly the same fw-Cu^{II} model previously employed to successfully fit the EXAFS spectrum at step #1 (pretreated in O_2) for quantitative analysis of the EXAFS spectrum at step #3 (oxidized in O_2 after reduction in NH_3/NO at 200 °C). The results of this test fit are detailed in Table **S4**.

Table S4. *Results of the test fit executed on the k^2-weighted FT-EXAFS spectrum of Cu-CHA oxidized in O_2 at 200 °C (step #3, red curve in Figure 3, main text) using as initial guess for the fitting procedure the same model of framework fw-Cu^{II} species presented in Table S2 and successfully used for EXAFS fitting on the pre-treated catalyst (step #1). Parameter values set in the fit (same as in Table S2) are underlined.* ***Unphysical values of the optimized parameters and of fitting errors are reported in red.***

Exp. – Step #3 Oxidized in O_2
Test EXAFS fitting model – fw-Cu^{II}

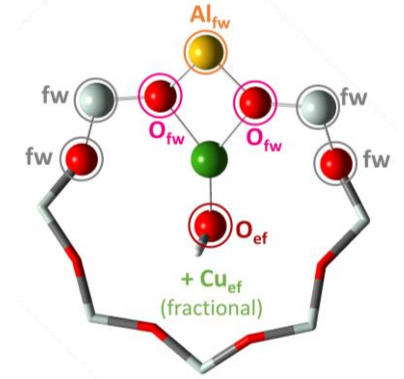

EXAFS parameters	***Best-fit values***
**R-space fitting range (Å)**	1.0-4.0
**$N°_{par}/N°_{ind}$**	10/19
**R_{factor}**	0.012
**S_0^2**	<u>0.9</u>
**ΔE (eV)**	-3 ± 3
**N_{O_ef} (Å)**	<u>1</u>
**N_{O_fw} (Å)**	<u>2</u>
**N_{Al_fw} (Å)**	<u>1</u>
**N_{Cu_ef} (Å)**	0.5 ± 0.3
**R_{O_ef} (Å)**	1.89 ± 0.04 (1.77)
**R_{O_fw} (Å)**	2.0 ± **0.1** (1.99)
**R_{Al_fw} (Å)**	**2.6 ± 0.9** (2.75)
**R_{Cu_ew} (Å)**	3.34 ± 0.05 (3.40)
**α_{fw}**	- 0.02 ± 0.01
**σ_O (Å^2)**	0.005 ± 0.006
**σ_{Al_fw} (Å^2)**	**0.1 ± 0.2**
**σ_{Cu_ef} (Å^2)**	<u>0.01</u>
**ss_{fw} (Å^2)**	0.019 ± 0.006

From the fitting results reported in Table **S4**, we note that the best fit curve is, mathematically speaking, in satisfactory agreement with the experimental one, resulting in a R-factor = 0.012.
However, this is achieved to the expense of the physical reliability and accuracy of the values of the optimized parameters. Such inconsistencies most severely affect the Cu-Al_{fw} coordination shell, representing a diagnostic contribution for all the fw-Cu^{II} species in the CHA framework. Strikingly, the fit restitutes σ_{Al_fw} = 0.1 ± 0.2 Å^2, more than one order of magnitude larger than typical expectation values, even in the presence of a high degree of structural heterogeneity (see e.g., Table **S2**: σ_{Al_fw} = 0.009 ± 0.003 Å^2). This is accompanied by an excessively high 0.15 Å contraction of R_{Al_fw} with respect to typical values found in DFT-optimized geometries for fw-coordinated Cu(II) species in CHA. Fitting errors associated to both R_{Al_fw} and σ_{Al_fw}, as well as to R_{O_fw} are one or two orders of magnitude higher than values found in well-behaved EXAFS fits for interatomic distances and DW factors, respectively.

This further underpins a global failure of the fitting model, thus supporting the spectroscopically-detectable diversity of Cu(II) species formed in Cu-CHA at step #1 and #3. The latter state, at step #3, is ultimately found to be not consistent with a fw-Cu^{II} species, paving the way to deeper structural analysis considering mobile $[Cu(II)_2(NH_3)_4O_2]^{2+}$ complexes, as presented in the following Section 2.4.

2.4 EXAFS analysis for oxidized state (step #3) considering $[Cu_2(NH_3)_4(O_2)]^{2+}$ models

2.4.1 Details on fitting strategy and shell model adopted for EXAFS analysis

EXAFS fits, reported in Table , have been performed taking into account the experimental k^2-weighted EXAFS spectrum of the investigated Cu-CHA catalyst oxidized at 200 °C in O_2, after reduction of the pre-treated sample in the NH_3 + NO mixture (red curve in Figure 3, main text). The DFT-optimized structures shown in Table for three candidate $[Cu_2(NH_3)_4(O_2)]^{2+}$ species have been used as initial guesses for the fitting procedure. The fit was performed on the experimental FT curve obtained by transforming the $k^2 \chi(k)$ curves in the 2.4 – 13.0 $Å^{-1}$ k-space range.

For the ***peroxo side-on*** and ***bis-μ-oxo*** geometries, a common (1.0 – 4.0) Å R-space range of fit was chosen while for the ***peroxo end-on*** complex, a larger R-space range (1.0 – 4.4) Å was selected, in accordance with the higher DFT Cu–Cu distance for this structure. This choice implied that the number of independent points ($N°_{ind}$) is equal to 19 for the ***peroxo side-on*** and ***bis-μ-oxo*** geometries, while for the ***peroxo end-on*** complex it is equal to 22.

Phases and backscattering amplitudes have been calculated by means of FEFF6.0, using the Arthemis software from the Demeter package.[4] The selected fitting models included all the single (SS) and multiple (MS) scattering paths contributing in the defined *R*-space ranges of fitting. An exception is represented by the SS and MS contributions coming from the H scatterers, whose scattering amplitude in the analysed models was found to be negligible in the fitting procedure. In order to limit the number of optimized variables, we refined all the SS and MS paths using the same passive amplitude reduction factor (S_0^2) and the same energy shift parameter (ΔE). The coordination numbers were fixed according to the related DFT-models.

In the employed models, all the SS paths were identified, and the related distances from the absorber were parametrized using an common guess value of radial shifts ΔR_x (with x=O^1, O^2, N and Cu, see Table) guessed starting from an initial value of 0 Å (i.e. no structural variation from the initial DFT distances). At the same time, the related Debye-Waller (DW) parameters were guessed on the basis of precedent fitting results.[24]

The identified SS paths can be then grouped as follow:

- SS involving first-shell bridging oxygen(s): Cu-O^1, with coordination number equal to 2 for the ***peroxo side-on*** and ***bis-μ-oxo*** geometries and equal to 1 for the ***peroxo end-on*** complex.
- SS involving first-shell nitrogen atoms of the two NH_3 molecules coordinated to the Cu absorber: Cu-N. In this case the DW factor, associated to the Cu-N paths, has been set to a value of 0.0041 $Å^2$ obtained by fitting with the $[Cu^I NH_3)_2]^+$ model structure the Cu K-edge EXAFS spectrum acquired at 200 °C under NO + NH_3 gas flow (dashed dark blue line in Figure 3, main text).
- SS involving second-shell bridging oxygen of the ***peroxo end-on*** configuration: Cu-O^2.
- SS involving the second Cu atom of the di-copper species.

Finally, all the MS paths have been parametrized, using the radial shifts ΔR_x proper of the SS paths on the basis of geometrical considerations. The DW parameters associated to each MS path were evaluated as the square root of the sum of squares of the DW terms of each paths involved in the process.

In the analysed models, as it is possible to see from Table , the number of identified fitting parameters is equal to 7 for the ***peroxo side-on*** and ***bis-μ-oxo*** geometries, while for the ***peroxo end-on*** configuration this number is increased up to 9, because of the second bridging oxygen O^2, closer to the second Cu atom. However, in both of cases, the number of retrieved fitting parameters remains always significantly lower than the number of employed independent points (i.e. ~ $2\Delta k \Delta R/\pi$) of 19 and 22.

The fit quality was evaluated considering the R-factor values and following this criterion: R-factor = 0: perfect agreement between experimental and theoretical curve; R-factor > 0.05: typical threshold for unsatisfactory fit.

2.4.2 Results from single-component EXAFS fits based on DFT-optimized candidate geometries

Table reports the fitting results using, as initial models, the DFT-optimized geometries of various $[Cu_2(NH_3)_4(O_2)]^{2+}$ complexes, namely ***peroxo side-on***, ***peroxo end-on*** and ***bis-μ-oxo*** geometries. How it is possible to observe, these last two complexes lead to unreliable results in terms of distances from the Cu absorber, showed in red in Table . In particular, EXAFS fit starting from ***bis-μ-oxo*** resulted in a large elongation of the Cu-Cu (~ +0.28 Å) and Cu-O^1 (~ +0.14 Å) distances, if compared to the respective DFT

models. In the same way, high variation of the Cu-Cu (~ +0.1 Å) and Cu-O^2 (~ +0.23 Å) DFT-optimized distances are found for the ***peroxo end-on*** configuration. For the fit staring from the ***peroxo end-on*** geometry, also certain DWs assumed unphysical values (highlighted in orange in Table), excessively small for the Cu-O^1 path (3× 10^{-4} Å^2) and too large for the Cu-Cu (0.02 Å^2) contribution.

Table S5. *Results of the fits executed on the k^2-weighted FT-EXAFS spectrum of Cu-CHA re-oxidized at 200 °C in O_2, after reduction of the pre-treated sample in the NH_3 + NO mixture (red curve in Figure 3, main text). The different models of $[Cu_2(NH_3)_4(O_2)]^{2+}$complex used as initial guesses for the fitting procedure are also shown, including two peroxo complexes with side-on (**peroxo side-on**) and end-on (**peroxo end-on**) configuration and a bis-μ-oxo complex (**bis-μ-oxo**). The averaged distances of the oxygens and nitrogen shells of atoms surrounding the Cu absorber are reported in brackets, "< >". Distances retrieved from the fitting procedure having a variation larger than 0.1 Å from the ones reported by the DFT optimized structures are reported in red. Unphysical values of DWs refined from the fit are indicated in orange. Colour code in the structures: **white**: hydrogen; **blue**: nitrogen; **red**: oxygen, **green:** copper. **Unphysical values of the optimized distances and DW factors are reported in red and orange, respectively.***

EXAFS fit parameters	*Peroxo side-on*	*Peroxo end-on*	*Bis-μ-oxo*
	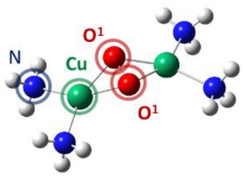	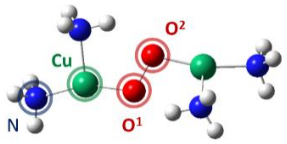	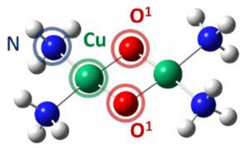
		**Best-fit values**	
**R-space fitting range (Å)**	1.0-4.0	1.0-4.4	1.0-4-0
**N°$_{par}$/N°$_{ind}$**	7/19	9/22	7/19
**R$_{factor}$**	0.021	0.004	0.011
**S_0^2**	0.82 ± 0.05	1.05±0.06	0.80 ± 0.03
**ΔE (eV)**	1 ± 1	2±1	1.7 ± 0.8
**<R$_{O1}$> (Å)**	1.911 ± 0.009 (1.95)	1.903 ± 0.008 (1.86)	**1.912 ± 0.006** (1.77)
**R$_{O2}$ (Å)**	-	**2.86 ± 0.04** (2.63)	-
**<R$_N$> (Å)**	2.06 ± 0.02 (2.03)	2.04 ± 0.01 (2.02)	**2.06 ± 0.01** (1.93)
**R$_{Cu}$ (Å)**	3.40 ± 0.05 (3.40)	**4.4 ± 0.2 (4.3)**	**2.93 ± 0.02** (2.64)
**σ$_{O1}$ (Å^2)**	0.0020 ± 0.0008	**0.0003 ± 0.0009**	0.0022 ± 0.0006
**σ$_{O2}$ (Å^2)**	-	0.008 ± 0.005	-
**σ$_N$ (Å^2)**	0.0041	0.0041	0.0041
**σ$_{Cu}$ (Å^2)**	0.014 ± 0.007	**0.02 ± 0.03**	0.012 ± 0.002

As it is possible to see from the first column of Table **S5**, among all the analysed models, the only reasonable fit, in terms of physical reliability of the optimised structural parameters, is obtained using the ***peroxo side-on geometry***, see Figure **S4**. In particular, the Cu-Cu distance is refined by the EXAFS fit to 3.40 ± 0.05 Å, fully consistent with the one provided by the DFT-optimised geometry. The R$_{factor}$ associated to the fitting procedure is representative of a good agreement with the experimental spectrum. However, in the 2.2 – 2.8 Å range, a slight mismatch between the experimental and the best fit curve can be appreciated (a possible explanation is provided below in Section 2.3).

It is interesting to note the higher complexity associated to the third-shell feature, sited around 3.1 Å (phase-uncorrected value). Herein, the fit shows that this feature cannot be attributed solely to the presence of the Cu-Cu path, but it is also shaped by relatively intense MS contributions. This result confirms the representation showed by the WT. In fact, as discussed in the main text, the second lobe projection of the wavelet in the *R*-space falls in the region of the FT-third shell. Here the WT shows two contributions: the first one, localised at low k-values in the k-range (2–4) Å^{-1}, typical of light scatterers (i.e. O and N) and the second one sited around k ~7 Å^{-1}, where the Cu backscattering amplitude $F(k)$ has its maximum value.

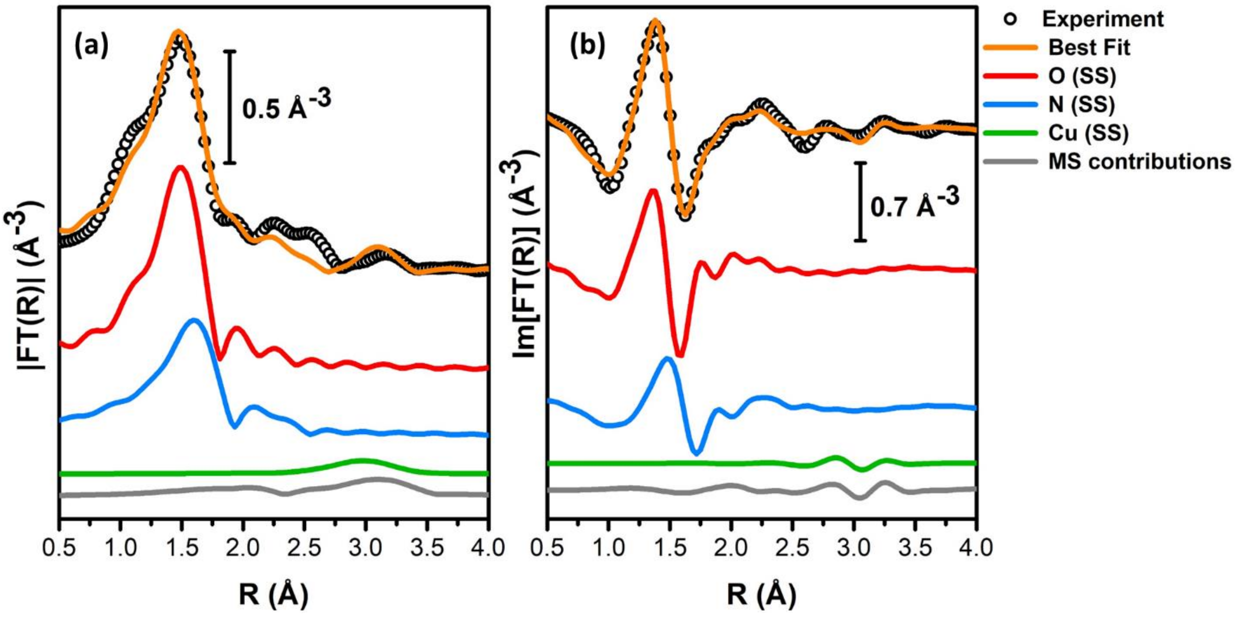

Figure S4. *Phase uncorrected, modulus (a) and imaginary part (b) of the experimental and best fit FT EXAFS spectra. The experimental data are shown as black open circles and the best fits with orange solid lines. The SS and MS contributions are reported as solid lines vertically translated for sake of clarity.*

2.4.3 Multi-component fit considering peroxo side-on $[Cu_2(NH_3)_4(O_2)]^{2+}$ and $[Cu^I(NH3)_2]^+$ complexes

Analysing the XANES spectrum of Cu-CHA oxidized at 200 °C in O_2 (red curve in Figure 3, main text), it is possible to observe the presence of a peak sited at ca. 8982.5 eV. This feature arises from the $1s \rightarrow 4p$ transition in Cu(I) centres and can be explained supposing that not all the $[Cu^I(NH_3)_2]^+$ formed upon exposure to the NO+NH_3 mixture have been oxidized, in presence of O_2, to ***peroxo side-on*** $[Cu_2(NH_3)_4(O_2)]^{2+}$ complexes. In order to quantify the amount of $[Cu^I(NH_3)_2]^+$ complexes still present in the sample after the oxygen treatment, a multi-component EXAFS fit has been performed, considering both the Cu(II) ***peroxo side-on*** $[Cu_2(NH_3)_4(O_2)]^{2+}$ and the $[Cu^I(NH_3)_2]^+$ complexes. In the fit, the amplitude reduction factor S_0^2 was set to the value of 0.9 by fitting the EXAFS reference of Cu(II)O, moreover, the Cu(I)-N distances (i.e. $\langle R_{N(CuI)} \rangle$) were set to a common value found fitting the spectrum of Cu-CHA reduced in the NH_3 + NO mixture at 200 °C using the[$Cu^I(NH_3)_2]^+$ model (how described for the σ_N in Section 4.1). In order to estimate the total amount of $[Cu^I(NH_3)_2]^+$, a new parameter *x*, expressing the fraction of $[Cu_2(NH_3)_4(O_2)]^{2+}$ in the zeolites cages, was introduced in the fit. In the case of ***peroxo side-on*** $[Cu_2(NH_3)_4(O_2)]^{2+}$ complex, the common S_0^2 term was then multiplied for the parameter *x*, while for $[Cu^I(NH_3)_2]^+$, a (1-*x*) factor was used. The result of the fit are reported in Table **S6** while Figure **S5** compares experimental and best-fit curve, as well as scaled contributions by the two structural components.

Table S6*. Best fit parameters obtained by two-component EXAFS fitting, including in the fitting model the structures of (1) side-on peroxo $[Cu_2(NH_3)_4(O_2)]^{2+}$ and (2) $[Cu^I(NH_3)_2]^+$ complex.*

EXAFS fit parameters	Best-fit values
**R-space fitting range (Å)**	1.0-4.0
**N°$_{par}$/N°$_{ind}$**	7/19
**R$_{factor}$**	0.020
**S_0^2**	0.9
**ΔE (eV)**	0 ± 1
**<R$_O$>(Å)**	1.916 ± 0.009 (1.95)
**<R$_N$>(Å)**	2.065 ± 0.009 (2.03)
**<R$_{N(CuI)}$>(Å)**	1.913
**R$_{Cu}$ (Å)**	3.39 ± 0.05 (3.40)
**σ_O (Å^2)**	0.0020 ± 0.0009
**σ_N (Å^2)**	0.0041
**σ_{Cu} (Å^2)**	0.013 ± 0.007
**x**	0.84 ± 0.08

From the fit output, it is possible to observe that the variation of the Cu-Cu distance, together with the new R_{factor} value, remain approximately the same as obtained by the single-component fit in Table **S5**. The fit refines the fraction of $[Cu^I(NH_3)_2]^+$ to the (16 ± 8) % of total Cu. This value is in good agreement with the intensity of the corresponding Cu(I) features observed in the corresponding XANES spectrum.

Nonetheless, a slight discrepancy between the best-fit curve and the experimental one persists in the 2.2–2.8 Å R-space range. We could tentatively connect this local lack in the fit quality to scattering contributions arising from zeolite framework atoms (Si, Al or even O), unaccounted in our model and likely present once the rather bulky ***peroxo side-on*** complex is hosted inside the CHA cages.

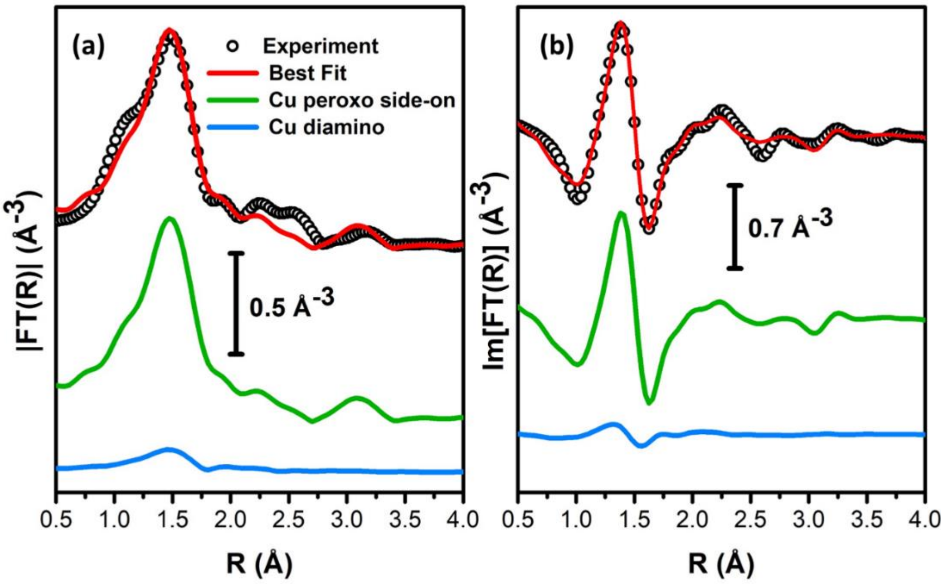

Figure S5. *k^2-weighted $\chi(k)$ phase uncorrected, modulus (a) and imaginary part (b) of the experimental and best multicomponent fit FT EXAFS spectra. The experimental data are shown as black open circles and the best fits with red solid lines. The contributions coming from the peroxo side-on $[Cu_2(NH_3)_4(O_2)]^{2+}$ and $[Cu(I)NH_3)_2]^+$ complexes weighted for their respective fractions (see Table) are reported as solid lines, vertically translated for sake of clarity.*

3 Wavelet Transform (WT) analysis of EXAFS spectra: methods and additional results

3.1 Relevance and methods for EXAFS WT analysis

It is possible to assert that if two or more groups of different atoms are localized at close distances around the absorber, their contributions in the direct space *R* overlap, becoming often indistinguishable.[25-27] This makes the classical Fourier transform (FT) analysis of the EXAFS spectrum rather uninformative in such cases. However, from a detailed analysis of the EXAFS equation, it is possible to observe that contributions, coming from the different scatterers surrounding the absorbing atom, are differently localized in the *k*-space, principally because of their related backscattering amplitude factor $F(k)$. This term depends strongly on the atomic number *Z*. In particular it is possible to observe that signals produced by heavier atoms, being characterized by large values of $F(k)$, are usually localized at higher wavenumbers than lighter atoms.[21, 27-28] This evidence can be exploited by a modern spectral processing technique called Wavelet Transform (WT), which can efficiently replace the *classical* FT representation of the EXAFS spectrum. WT is able to provide a 2D representation of the EXAFS, revealing, in this way, the signal features both in *k* and *R*-spaces simultaneously.
The WT of a general k^n-weighted EXAFS spectrum is defined as:

$$w(a,b) = \frac{1}{\sqrt{a}} \int_{-\infty}^{+\infty} dk' \, k'^n \chi(k') \psi^* \left(\frac{k'-b}{a} \right) \quad \textbf{(S1)}$$

This equation can be seen as the inner product between the k^n-weighted EXAFS spectrum $\chi(k)$ and a defined window function ψ, called *mother wavelet* or simply wavelet (where ψ^* denotes the complex conjugate of ψ), which must decay at zero for higher values of $|k'|$. Here, the signal $\chi(k)$ is analysed through a set of train-waves (wavelets) that are shifted by *b* units in the *k*-space and distorted by a factor *a* in order to take account of the local frequencies of the signal.[29-30] The variables *a* and *b* are connected to *k*- and *R*-space by the following relations: $a = \eta/2R$ and $b = k$.
In this work, the Morlet wavelet expressed in the following form was used:

$$\psi(k) = \frac{1}{\sqrt{2\pi}\sigma} \exp\,(i\eta k) \exp\,(-k^2/2\sigma^2) \quad \text{(S2)}$$

Where *i* denotes the complex unit while η and σ are two parameters regulating the wavelet resolution in *R* and *k* spaces.[25, 31] The selection of an appropriate set of these two parameters is critical and must be optimized in order to have the best visualization of the desired spectral features. In fact, it is worth mentioning that the WT distributes the signal information over *k*-*R* cells, usually named as *uncertainty* or *Heisenberg boxes* [31], which for the Morlet wavelet can be expressed as:

$$\left[k \pm \frac{\eta\sigma}{\sqrt{2}R} \right] \times \left[R \pm \frac{R}{\sqrt{2}\eta\sigma} \right] \quad \textbf{(S3)}$$

On this basis, it is possible to see that the resolution in *k* and *R* space are inversely proportional; this means that a good resolution in *k*-space always implies a loss of information in the related *R*-space, and vice versa.

3.2 Comparative overview of in situ EXAFS spectra by conventional visualization in k-space and R-space

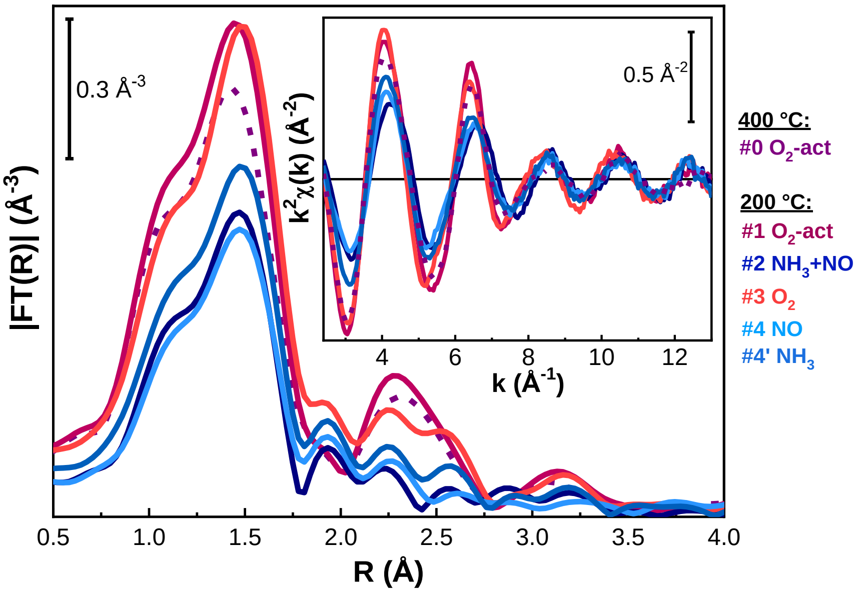

Figure S6. *Comparative overview of conventional Fourier Transform (FT) EXAFS spectra collected after sample pre-treatment in O_2 at 400 °C, cooling in O_2 down to 200 °C, and after the different investigated reactivity steps at 200 °C. The inset reports the related k^2-weighted $\chi(k)$ spectra. The FT curves have been obtained transforming the k^2 $\chi(k)$ curves in the 2.4 – 13.0 Å^{-1} k-space range This range was selected using a Hanning window with a symmetric decay parameter dk fixed to 1.*

3.3 Full-range Wavelet Transform representation of in situ EXAFS spectra

We applied the WT on the set of EXAFS spectra collected after pre-treatment in O_2 as well as after the investigated SCR-related reactivity steps. A comparative overview of relevant set of EXAFS spectra is reported in Figure **S6**. Due to its sensitivity to the chemical nature of the scatterers surrounding the absorbing atom,[27] WT can be exploited to unambiguously assess the presence of Cu-Cu scattering contributions for each spectrum showed in Figure **S6**. Cu-Cu distances in multi-copper moieties possibly forming after high-temperature pre-treatment in O_2 (steps #0 and #1 in Figure **S6**) and during the SCR-related reactivity steps (step #2-4#' in Figure **S6**) typically falls around 3.5 Å range. We chose, for all the wavelet representation, the following set of parameters: $\sigma = 1$ and $\eta = 7$. This choice aims at satisfying the condition of *optimal resolution* at a given distance of interest, as described by Funke et al.,[32] where it is showed that the best discrimination is reached for $\sigma = 1$ and $\eta = 2R_{opt}$, where R_{opt} is the real backscattering distance.

The full-rage WT representations for all the selected EXAFS spectra showed in **S6** are reported below in Figures S7-S9.

The conformation of the high-R lobes of WTs, extending in the region Δk (0-10) Å^{-1} × ΔR (2.2-4) Å is discussed in detail in the main text (Section 3.1.3). Herein, we comment on the first lobes WT maps, located in the Δk (0-14) Å^{-1} × ΔR (0.5-2.0) Å range.

In case of WT plots of the EXAFS spectra showed in Figure S7, the first WT lobe is principally determined by the single scattering contributions stemming from framework (O_{fw}) and extra-framework (O_{ef}) oxygen atoms in the first Cu coordination shell, as described in ref.[21] WT plots reported in Figure S8 are related to the formation of monomeric Cu(I) (i.e. $[Cu(I)NH_3)_2]^+$) and dimeric Cu(II) sites (i.e. $[Cu_2(NH_3)_4(O_2)]^{2+}$). Here the first, low-R WT lobe arises from the nitrogen and oxygen (only for the Cu-dimer) atoms always sited in the first coordination shell of Cu centers. A similar description can be associated for the first lobe of WT graphs in Figure S9, arising either from N single scattering contributions in mobile Cu(I)/Cu(II) amino-complexes (Figure S9a, step #4') or from both N and O_{fw} single-scattering contribution (Figure S9b, step #4) in framework-coordinated $O_{fw}Cu(I)NH_3$ species.[33]

It is worth noting that the evident elongation of the first lobe towards large *k*-values should not be associated to the presence of heavier scattering atoms, but rather explained introducing the concept of spectral broadening. In fact, as demonstrated by Timoshenko et al.[27] if, for low *R*-values, the relation $\frac{1}{a} \ll \eta$ is satisfied, the wavelet

lobes are distorted in k-space. This is the case of first lobes of the analyzed wavelet where the low R range allow to satisfy the condition showed before.

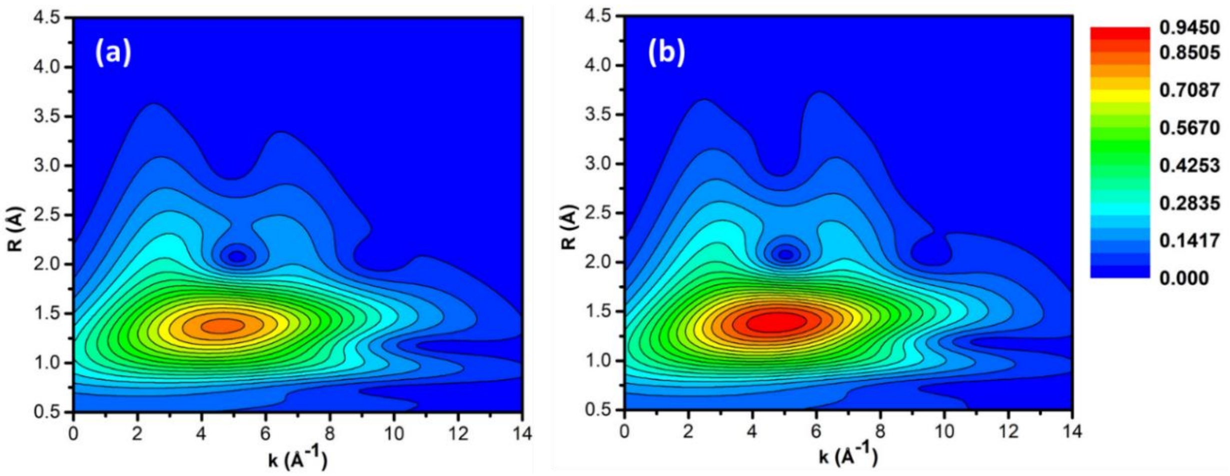

Figure S7. *Moduli of EXAFS-WT for spectra referring to steps **#0** (a) and **#1** (b) in Figure **S6**.*

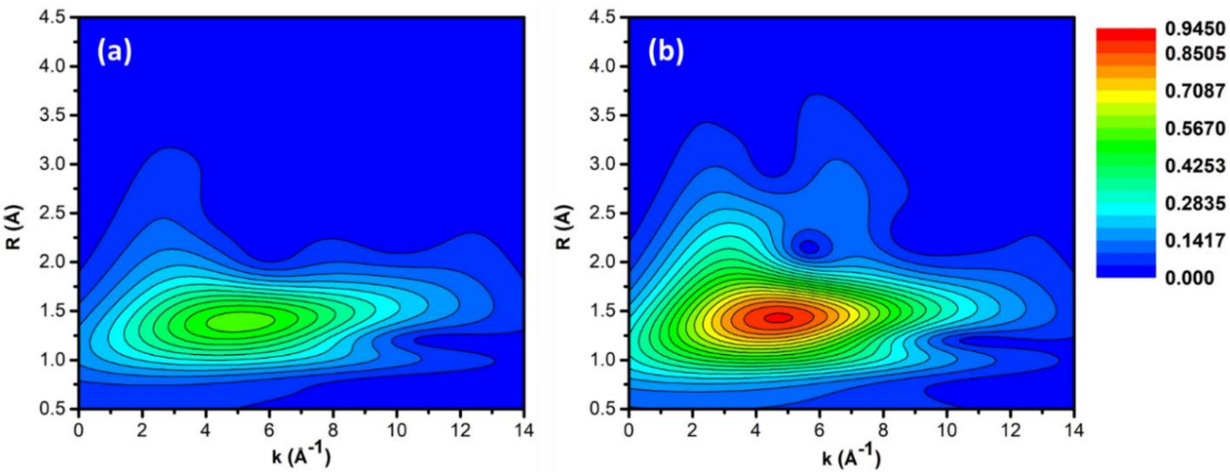

Figure S8. *Moduli of EXAFS-WT for spectra referring to steps **#2** (a) and **#3** (b) in Figure **S6**.*

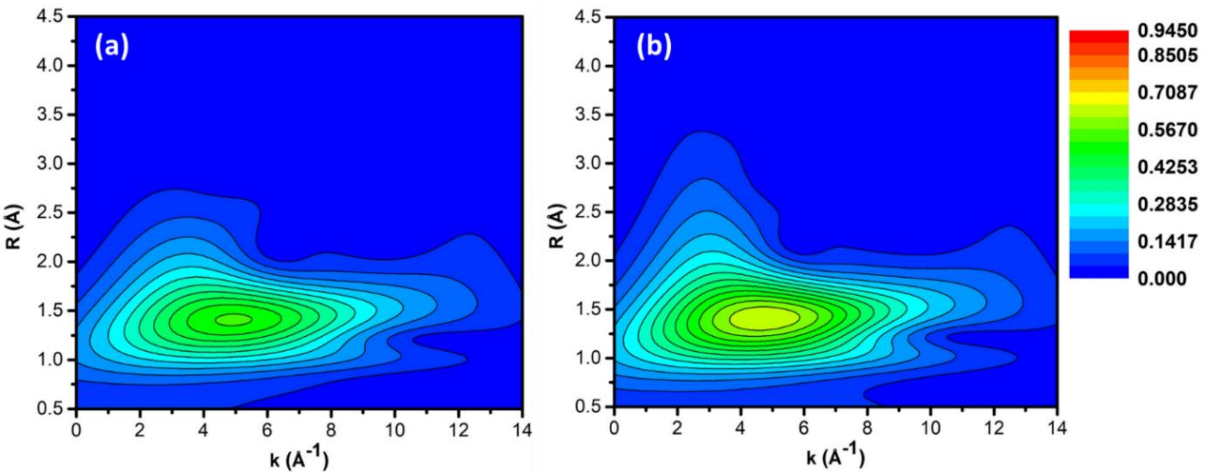

Figure S9. *Moduli of EXAFS-WT for spectra referring to steps **#4** (a) and **#4'** (b) of Figure **S6**.*

4 Additional *in situ* spectroscopy results

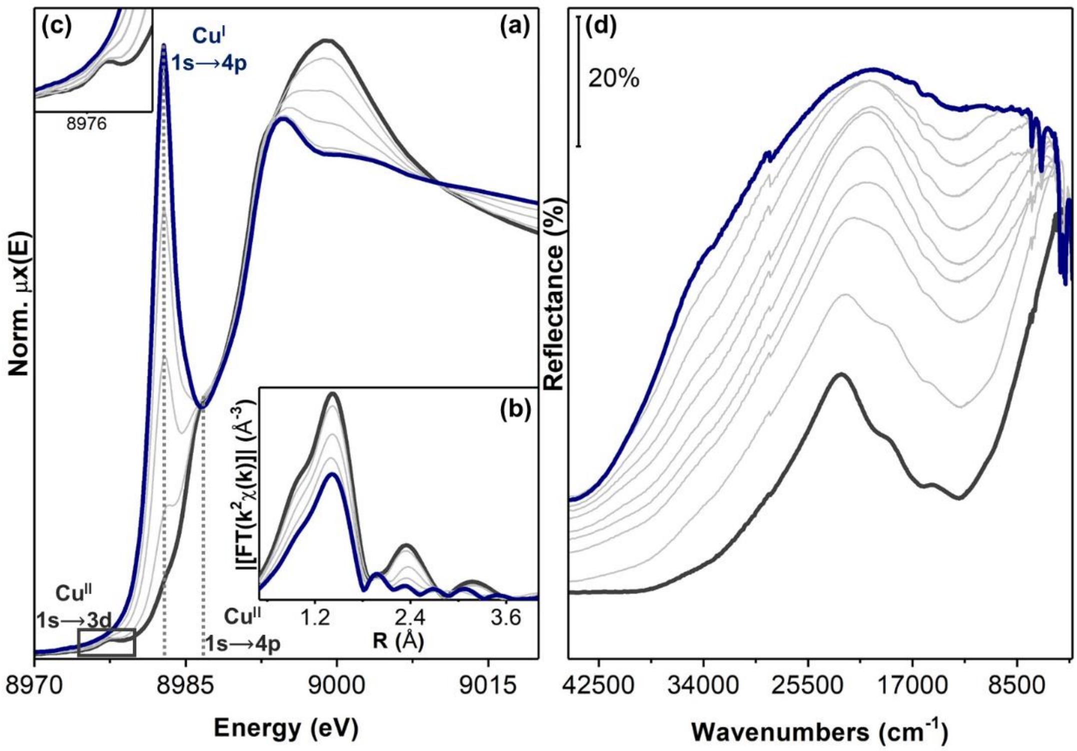

Figure S10. *Evolution of the operando XAS and UV-Vis-NIR DR spectra of Cu-CHA during exposure to 1000 ppm NO/1000 ppm NH_3 He balanced at 200 °C after pre-treatment in 100% O_2 at 400 °C. (a): Cu K-edge XANES spectra; (b) k^2-weighted $k^2\chi(k)$ EXAFS curves; (c) Magnification of Cu^{II} $1s \rightarrow 3d$ transition; (d) evolution of UV-Vis-NIR Diffuse Reflectance spectra. Colour code: dark grey thick line: spectrum after pre-treatment in O_2 at 200 °C; dark blue thick line: after NO/NH_3/He exposure at 200 °C; grey thin lines: intermediates.*

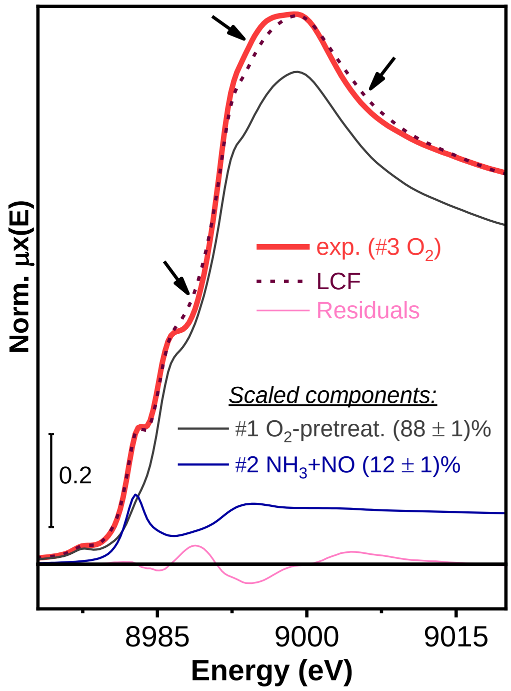

Figure S11. *Test XANES LCF aimed at reproducing the XANES spectrum of the Cu-CHA catalyst at step #3 (oxidized in O_2 at 200 °C) using as references for Cu^{II} and Cu^I components the XANES spectra of the same catalyst collected at step #1 (pretreated in O_2, measured at 200 °C) and at step #2 (reduced in NO/NH_3 at 200 °C), respectively. The arrows highlight spectral ranges where larger mismatch between experimental and LCF curve is observed, corresponding to well-defined positive and negative peaks of the residuals (pink solid line).*

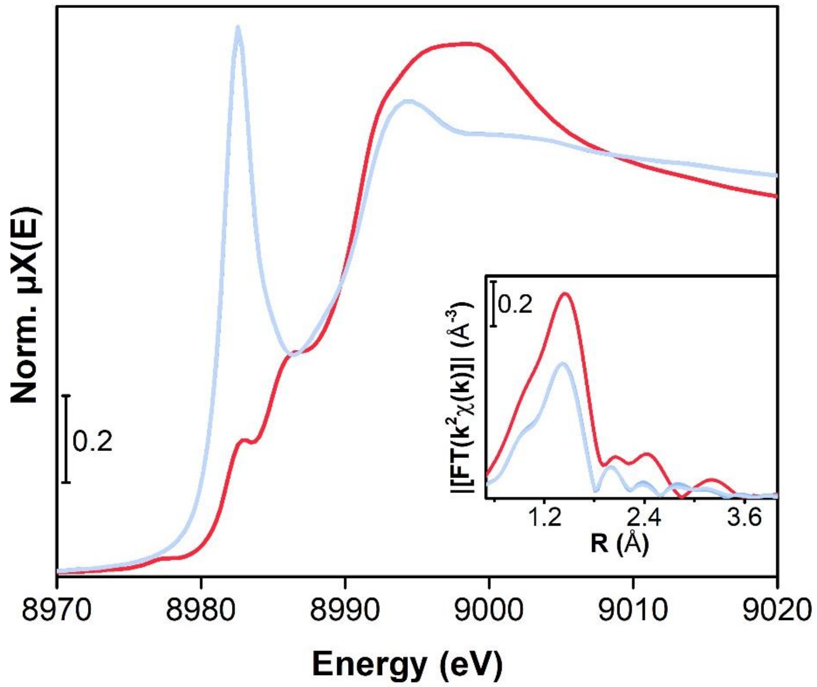

Figure S12. *Evolution of the operando XAS of Cu-CHA during exposure to 1000 ppm NO/1000 ppm NH_3 He balanced at 200 °C after oxidation in 10% O_2/He at 200 °C. (a): Cu K-edge XANES spectra; (b) k^2-weighted $k^2\chi(k)$ EXAFS curves; Colour code: red line: spectrum after exposure to 10% O_2 at 200 °C; light grey line: after NO/NH_3/He exposure at 200 °C.*

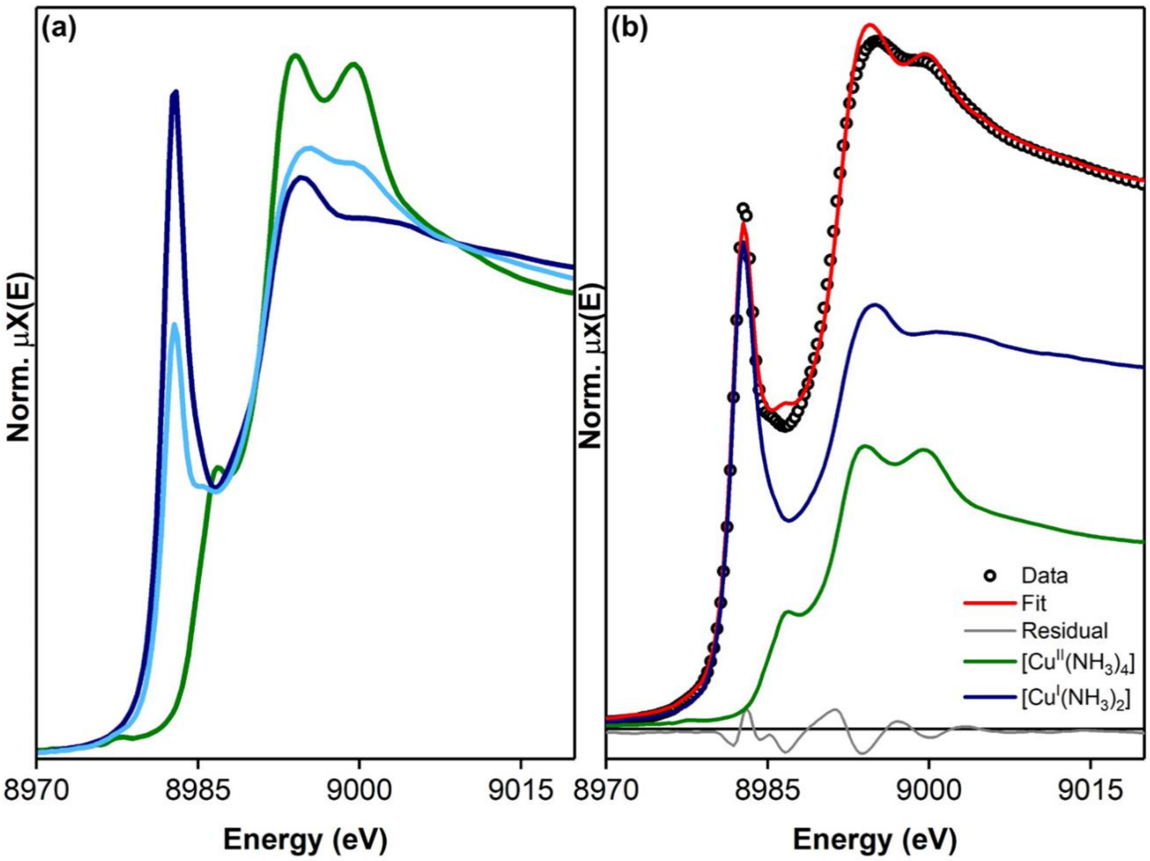

Figure S13. *(a): Cu K-edge XANES spectra of [$Cu^{II}(NH_3)_4$] (green curve), of [$Cu^{I}(NH_3)_2$] (dark blue curve) and after 1000 ppm NH_3/He exposure at 200 °C (light blue curve); (b) Result for the Linear Combination Fit.*

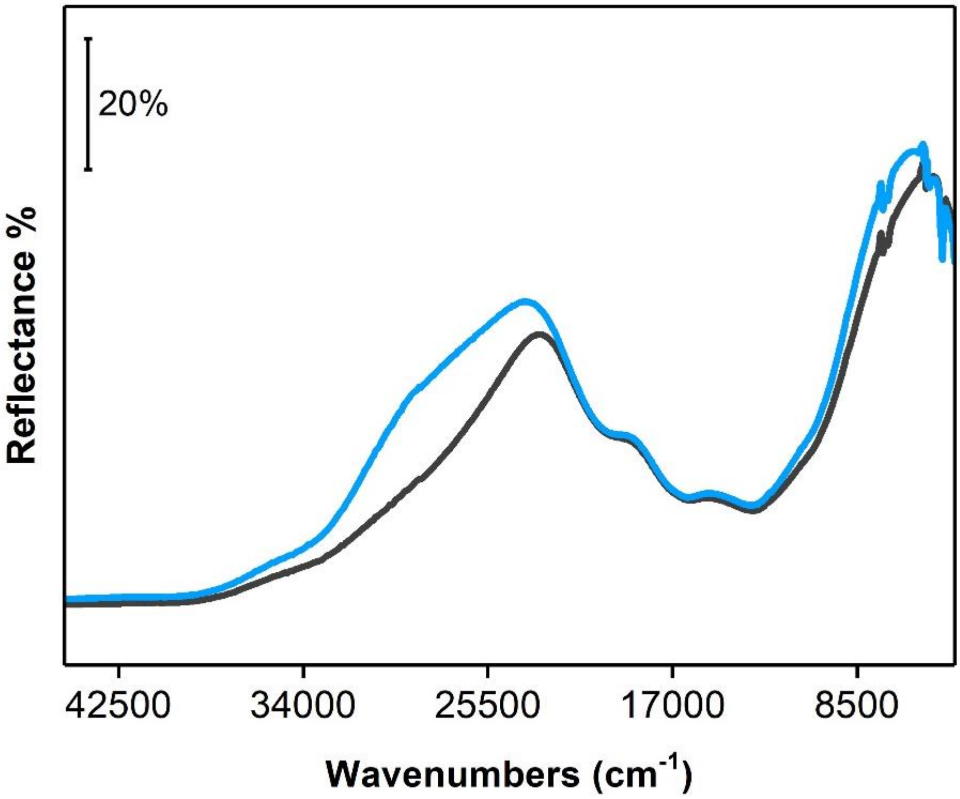

Figure S14. *UV-Vis-NIR Diffuse Reflectance spectra of Cu-CHA after exposure to 1000 ppm NO/He (blue curve) and of Cu-CHA after pre-treatment in 100% O_2 at 400 °C (dark grey curve). Both spectra are measured at 200 °C*

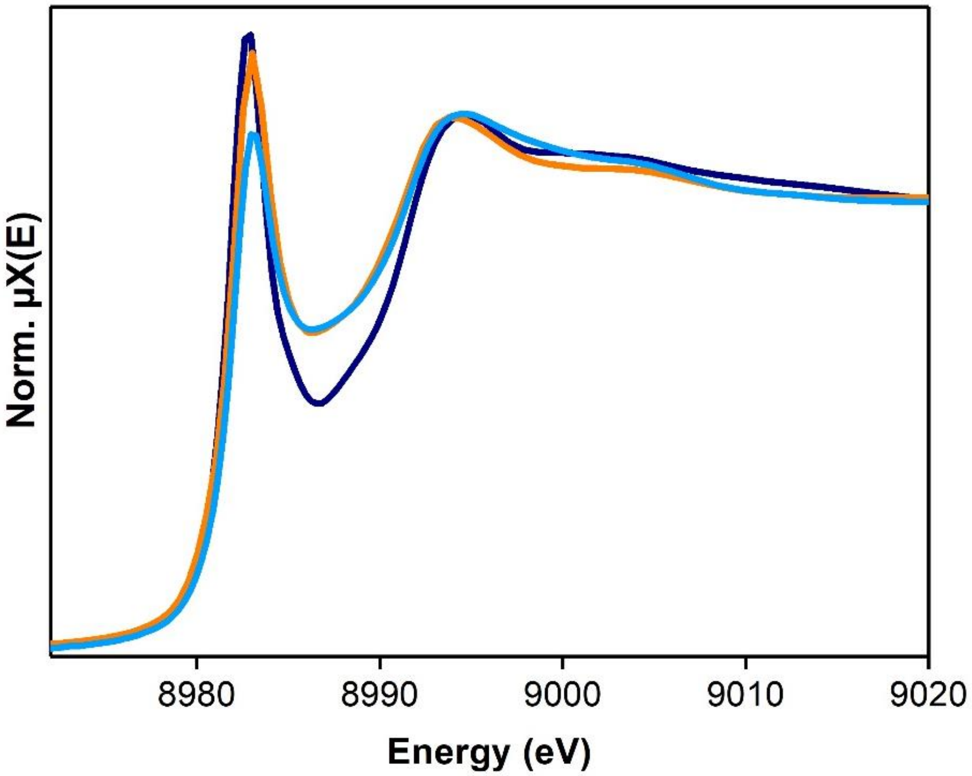

Figure S15*. Cu K-edge XANES spectra of $[Cu^{I}(NH_3)_2]$ (dark blue curve), of $Z[Cu^{I}(NH_3)]$ (orange curve) and of Cu-CHA after exposure to 1000 ppm NO/He at 200 °C (blue curve).*

5 Mass Spectrometry results obtained during *in situ* XAS experiments

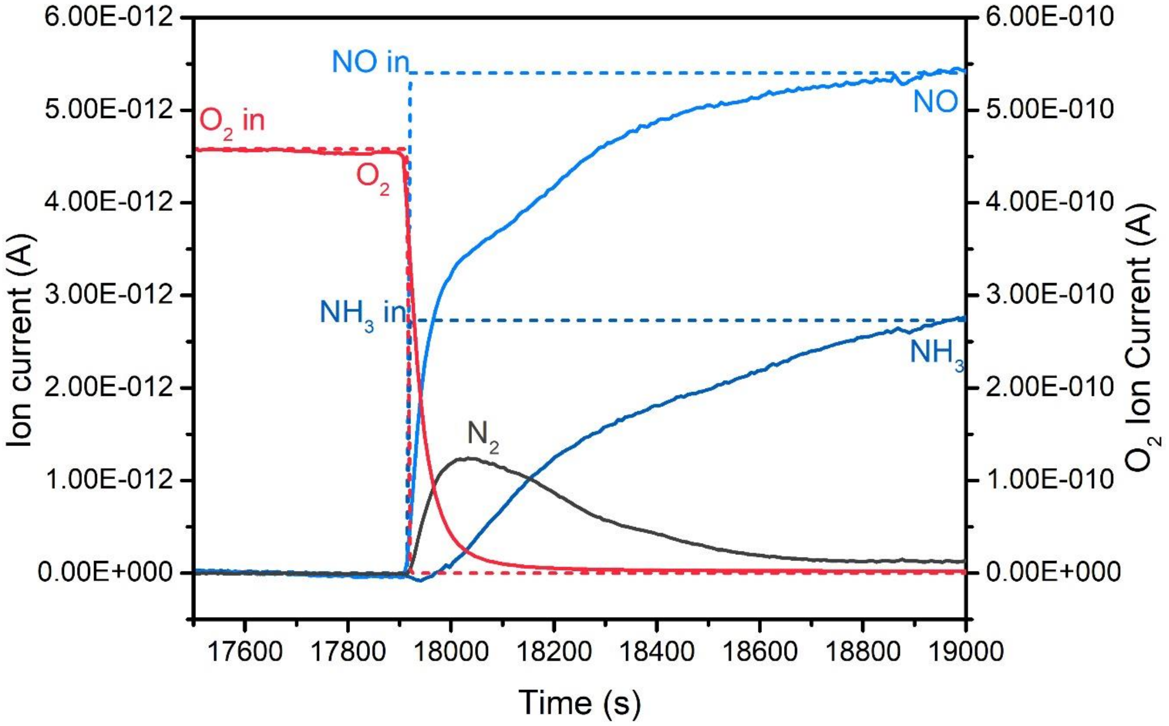

Figure S16. *Mass Spectrometry data collected during NO/NH$_3$ reaction with the mobile [Cu$_2$(O$_2$)(NH$_3$)$_4$]$^{2+}$ complex.*

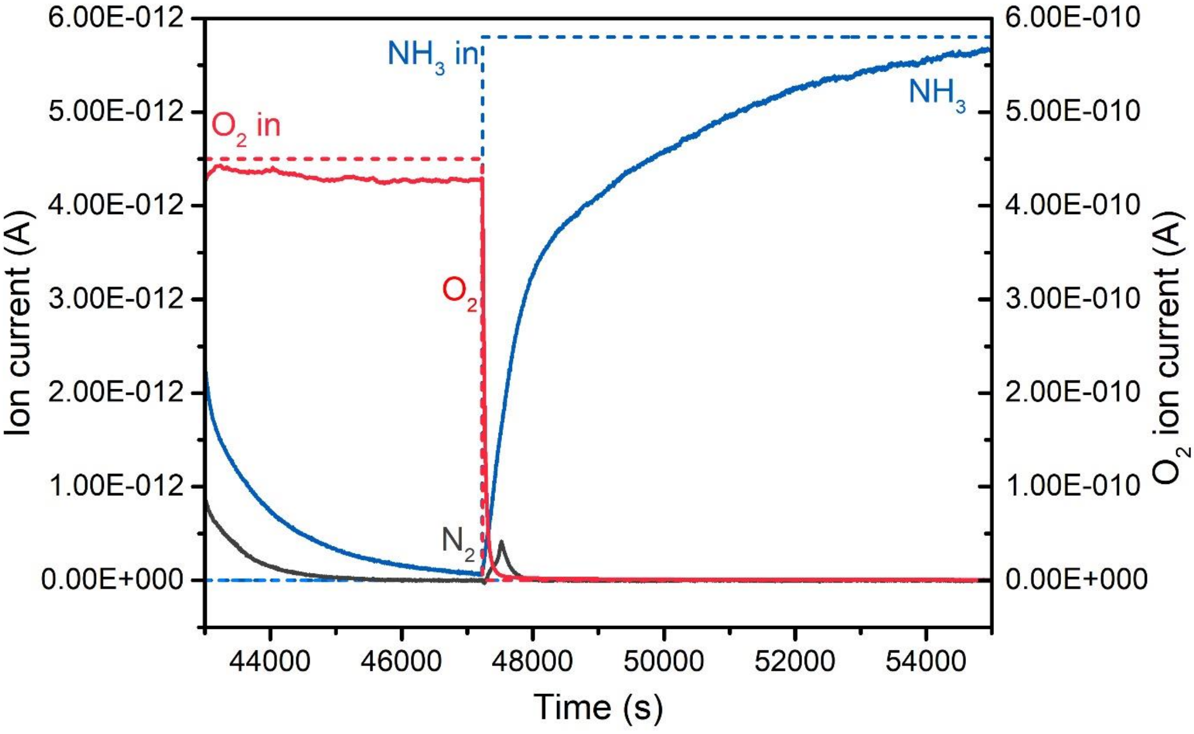

Figure S17. *Mass Spectrometry data collected during NH$_3$ reaction with the mobile [Cu$_2$(O$_2$)(NH$_3$)$_4$]$^{2+}$ complex.*

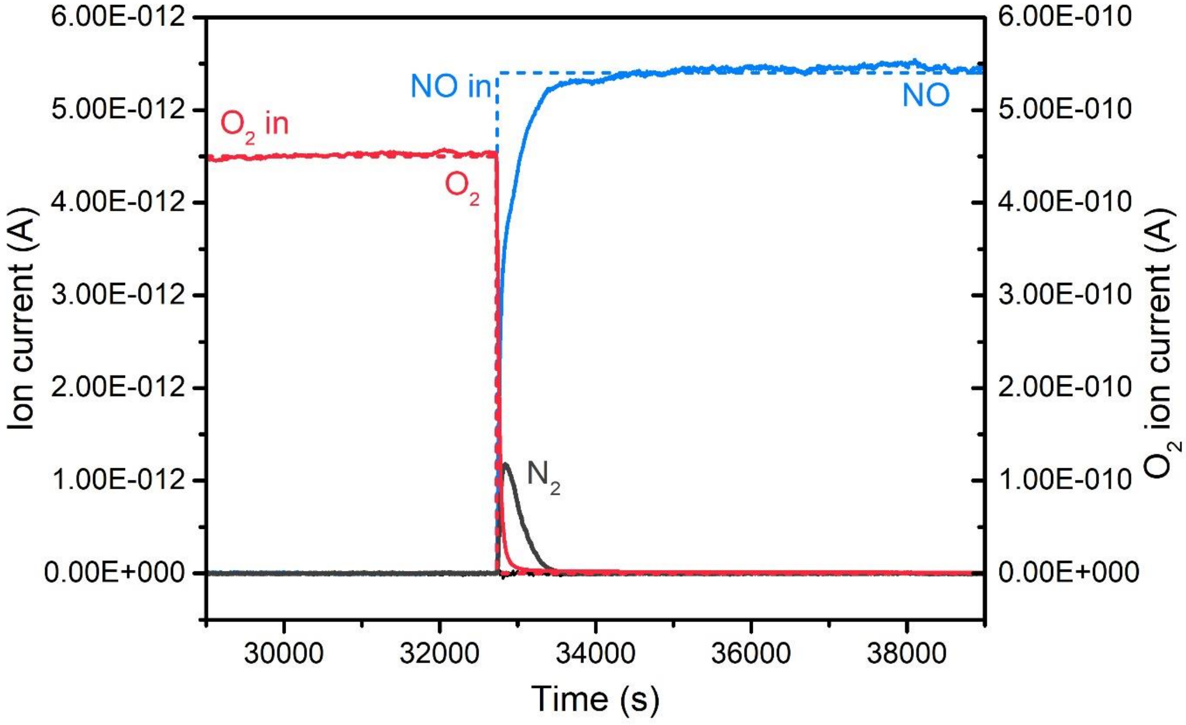

Figure S18. *Mass Spectrometry data collected during NO reaction with the mobile* $[Cu_2(O_2)(NH_3)_4]^{2+}$ *complex.*

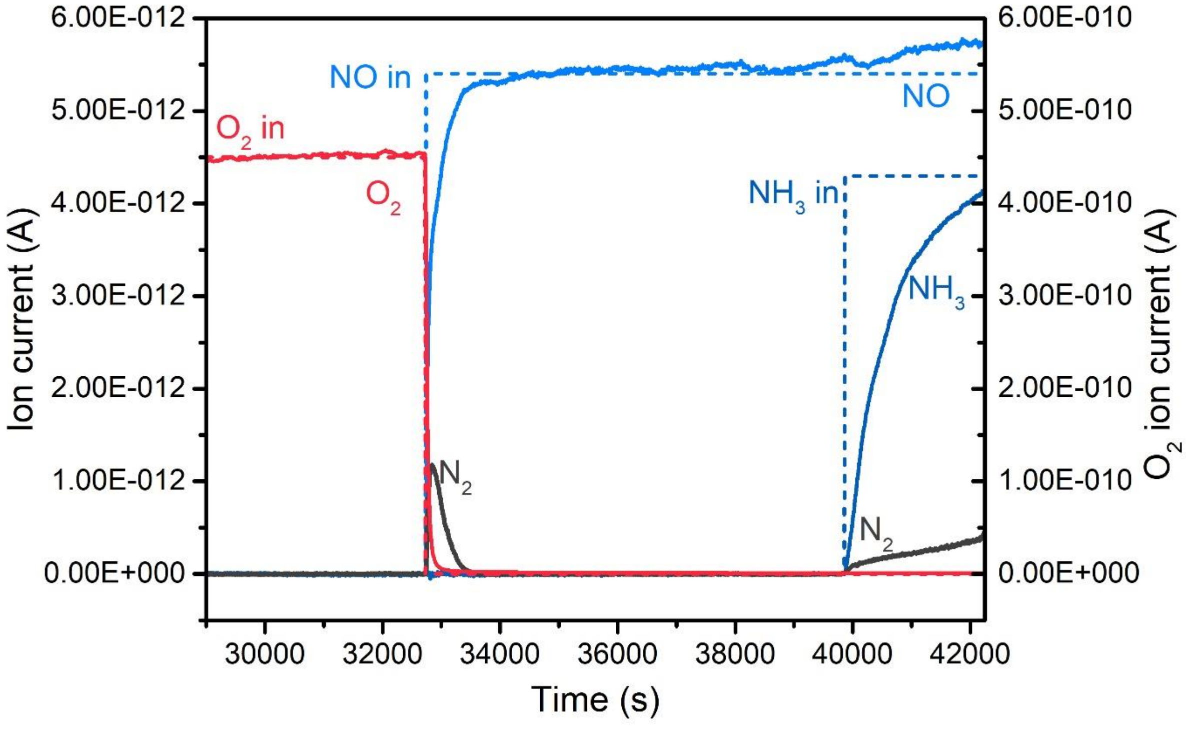

Figure S19. *Mass Spectrometry data collected when adding* NH_3 *to the NO feed after step #4.*

References

1. Mathon, O.; Beteva, A.; Borrel, J.; Bugnazet, D.; Gatla, S.; Hino, R.; Kantor, I.; Mairs, T.; Munoz, M.; Pasternak, S.; Perrin, F.; Pascarelli, S., The time-resolved and extreme conditions XAS (TEXAS) facility at the European Synchrotron Radiation Facility: the general-purpose EXAFS bending-magnet beamline BM23. *J. Synchrot. Radiat.* **2015,** *22*, 1548-1554.
2. Bellet, D.; Gorges, B.; Dallery, A.; Bernard, P.; Pereiro, E.; Baruchel, J., A 1300 K furnace for in situ X-ray microtomography. *J. Appl. Crystallogr.* **2003,** *36*, 366-367.
3. Bordiga, S.; Groppo, E.; Agostini, G.; van Bokhoven, J. A.; Lamberti, C., Reactivity of surface species in heterogeneous catalysts probed by in situ X-ray absorption techniques. *Chem. Rev.* **2013,** *113* (3), 1736-1850.
4. Ravel, B.; Newville, M., ATHENA and ARTEMIS: Interactive graphical data analysis using IFEFFIT. *Phys. Scr.* **2005,** *T115*, 1007-1010.
5. Cramer, C. J.; Kinal, A.; Wloch, M.; Piecuch, P.; Gagliardi, L., Theoretical characterization of end-on and side-on peroxide coordination in ligated Cu2O2 models. *J. Phys. Chem. A* **2006,** *110* (40), 11557-11568.
6. Cramer, C. J.; Wloch, M.; Piecuch, P.; Puzzarini, C.; Gagliardi, L., Theoretical models on the Cu2O2 torture track: Mechanistic implications for oxytyrosinase and small-molecule analogues. *J. Phys. Chem. A* **2006,** *110* (5), 1991-2004.
7. Paolucci, C.; Khurana, I.; Parekh, A. A.; Li, S. C.; Shih, A. J.; Li, H.; Di Iorio, J. R.; Albarracin-Caballero, J. D.; Yezerets, A.; Miller, J. T.; Delgass, W. N.; Ribeiro, F. H.; Schneider, W. F.; Gounder, R., Dynamic multinuclear sites formed by mobilized copper ions in NOx selective catalytic reduction. *Science* **2017,** *357* (6354), 898-+.
8. Chen, L.; Janssens, T. V. W.; Gronbeck, H., A comparative test of different density functionals for calculations of NH3-SCR over Cu-Chabazite. *Phys. Chem. Chem. Phys.* **2019,** *21* (21), 10923-10930.
9. Peccati, F.; Laplaza, R.; Contreras-García, J., Overcoming Distrust in Solid State Simulations: Adding Error Bars to Computational Data. *J. Phys. Chem. C* **2019,** *123* (8), 4767-4772.
10. Zhao, Y.; Truhlar, D. G., A new local density functional for main-group thermochemistry, transition metal bonding, thermochemical kinetics, and noncovalent interactions. **2006,** *125* (19), 194101.
11. Zhao, Y.; Truhlar, D. G., Density Functional for Spectroscopy: No Long-Range Self-Interaction Error, Good Performance for Rydberg and Charge-Transfer States, and Better Performance on Average than B3LYP for Ground States. *The Journal of Physical Chemistry A* **2006,** *110* (49), 13126-13130.
12. Zhao, Y.; Truhlar, D. G., The M06 suite of density functionals for main group thermochemistry, thermochemical kinetics, noncovalent interactions, excited states, and transition elements: two new functionals and systematic testing of four M06-class functionals and 12 other functionals. *Theoretical Chemistry Accounts* **2008,** *120* (1), 215-241.
13. Frisch, M. J.; Trucks, G. W.; Schlegel, H. B.; Scuseria, G. E.; Robb, M. A.; Cheeseman, J. R.; Scalmani, G.; Barone, V.; Mennucci, B.; Petersson, G. A.; al., e., **2013,** *Gaussian, Inc. Wallingford, CT*.
14. Grimme, S.; Antony, J.; Ehrlich, S.; Krieg, H., A consistent and accurate ab initio parametrization of density functional dispersion correction (DFT-D) for the 94 elements H-Pu. *J. Chem. Phys.* **2010,** *132* (15).
15. Weigend, F., Accurate Coulomb-fitting basis sets for H to Rn. *Phys. Chem. Chem. Phys.* **2006,** *8* (9), 1057-1065.
16. Miertuš, S.; Scrocco, E.; Tomasi, J., Electrostatic interaction of a solute with a continuum. A direct utilizaion of AB initio molecular potentials for the prevision of solvent effects. *Chemical Physics* **1981,** *55* (1), 117-129.
17. Billaud, G.; Demortier, A., Dielectric constant of liquid ammonia from -35 to + 50.deg. and its influence on the association between solvated electrons and cation. *J. Phys. Chem.* **1975,** *79* (26), 3053-3055.
18. Borfecchia, E.; Lomachenko, K. A.; Giordanino, F.; Falsig, H.; Beato, P.; Soldatov, A. V.; Bordiga, S.; Lamberti, C., Revisiting the nature of Cu sites in the activated Cu-SSZ-13 catalyst for SCR reaction. *Chem. Sci.* **2015,** *6* (1), 548-563.
19. Pappas, D. K.; Borfecchia, E.; Dyballa, M.; Pankin, I. A.; Lomachenko, K. A.; Martini, A.; Signorile, M.; Teketel, S.; Arstad, B.; Berlier, G.; Lamberti, C.; Bordiga, S.; Olsbye, U.; Lillerud, K. P.; Svelle, S.; Beato, P., Methane to Methanol: Structure Activity Relationships for Cu-CHA. *J. Am. Chem. Soc.* **2017,** *139* (42), 14961-14975.

20. Li, H.; Paolucci, C.; Khurana, I.; Wilcox, L.; Goltl, F.; Albarracin-Caballero, J. D.; Shih, A. J.; Ribeiro, F. H.; Gounder, R.; Schneider, W. F., Consequences of exchange-site heterogeneity and dynamics on the UV-visible spectrum of Cu-exchanged SSZ-13. *Chem. Sci.* **2019,** *10* (8), 2373-2384.
21. Pankin, I. A.; Martini, A.; Lomachenko, K. A.; Soldatov, A. V.; Bordiga, S.; Borfecchia, E., Identifying Cu-oxo species in Cu-zeolites by XAS: A theoretical survey by DFT-assisted XANES simulation and EXAFS wavelet transform. *Catal. Today* **2020,** *345*, 125-135.
22. Vilella, L.; Studt, F., The Stability of Copper Oxo Species in Zeolite Frameworks. *Eur. J. Inorg. Chem.* **2016,** *2016* (10), 1514-1520.
23. Ipek, B.; Wulfers, M. J.; Kim, H.; Goltl, F.; Hermans, I.; Smith, J. P.; Booksh, K. S.; Brown, C. M.; Lobo, R. F., Formation of Cu2O2 (2+) and Cu2O (2+) toward C-H Bond Activation in Cu-SSZ-13 and Cu-SSZ-39. *ACS Catal.* **2017,** *7* (7), 4291-4303.
24. Martini, A.; Borfecchia, E.; Lomachenko, K. A.; Pankin, I. A.; Negri, C.; Berlier, G.; Beato, P.; Falsig, H.; Bordiga, S.; Lamberti, C., Composition-driven Cu-speciation and reducibility in Cu-CHA zeolite catalysts: a multivariate XAS/FTIR approach to complexity. *Chem. Sci.* **2017,** *8* (10), 6836-6851.
25. Funke, H.; Scheinost, A. C.; Chukalina, M., Wavelet analysis of extended x-ray absorption fine structure data. *Phys. Rev. B* **2005,** *71* (9), 7.
26. Munoz, M.; Argoul, P.; Farges, F., Continuous Cauchy wavelet transform analyses of EXAFS spectra: A qualitative approach. *Am. Mineral.* **2003,** *88* (4), 694-700.
27. Timoshenko, J.; Kuzmin, A., Wavelet data analysis of EXAFS spectra. *Comput. Phys. Commun.* **2009,** *180* (6), 920-925.
28. Lomachenko, K. A.; Martini, A.; Pappas, D. K.; Negri, C.; Dyballa, M.; Berlier, G.; Bordiga, S.; Lamberti, C.; Olsbye, U.; Svelle, S.; Beato, P.; Borfecchia, E., The impact of reaction conditions and material composition on the stepwise methane to methanol conversion over Cu-MOR: An operando XAS study. *Catal. Today* **2019,** *336*, 99-108.
29. Martini, A.; Pankin, I. A.; Marsicano, A.; Lomachenko, K. A.; Borfecchia, E., Wavelet analysis of a Cu-oxo zeolite EXAFS simulated spectrum. *Radiat. Phys. Chem.* **2019**, 108333.
30. Soldatov, M. A.; Martini, A.; Bugaev, A. L.; Pankin, I.; Medvedev, P. V.; Guda, A. A.; Aboraia, A. M.; Podkovyrina, Y. S.; Budnyk, A. P.; Soldatov, A. A.; Lamberti, C., The insights from X-ray absorption spectroscopy into the local atomic structure and chemical bonding of Metal-organic frameworks. *Polyhedron* **2018,** *155*, 232-253.
31. Penfold, T. J.; Tavernelli, I.; Milne, C. J.; Reinhard, M.; El Nahhas, A.; Abela, R.; Rothlisberger, U.; Chergui, M., A wavelet analysis for the X-ray absorption spectra of molecules. *J. Chem. Phys.* **2013,** *138* (1), 7.
32. Funke, H.; Chukalina, M.; Scheinost, A. C., A new FEFF-based wavelet for EXAFS data analysis. *J. Synchrot. Radiat.* **2007,** *14*, 426-432.
33. Borfecchia, E.; Negri, C.; Lomachenko, K. A.; Lamberti, C.; Janssens, T. V. W.; Berlier, G., Temperature-dependent dynamics of NH3-derived Cu species in the Cu-CHA SCR catalyst. *React. Chem. Eng.* **2019,** *4* (6), 1067-1080.
